# An Identification and Characterization of the Axolotl (*Ambystoma mexicanum, Amex*) Telomerase Reverse Transcriptase (Amex TERT)

**DOI:** 10.3390/genes13020373

**Published:** 2022-02-18

**Authors:** Sina Springhetti, Vesna Bucan, Christina Liebsch, Andrea Lazaridis, Peter Maria Vogt, Sarah Strauß

**Affiliations:** 1 Department of Plastic, Aesthetic, Hand and Reconstructive Surgery, Hannover Medical School, 30625 Hannover, Germany; bucan.vesna@mh-hannover.de (V.B.); liebsch.christina@mh-hannover.de (C.L.); lazaridis.andrea@mh-hannover.de (A.L.); vogt.peter@mh-hannover.de (P.M.V.); strauss.sarah@mh-hannover.de (S.S.); 2 Department of Oral and Maxillofacial Surgery, Diakovere Henriettenstift, 30171 Hannover, Germany

**Keywords:** limb regeneration, axolotl, telomerase, telomerase reverse transcriptase (TERT), senescence, telomere biology, dedifferentiation

## Abstract

The Mexican axolotl is one of the few vertebrates that is able to replace its lost body parts during lifespan. Due to its remarkable regenerative abilities, the axolotl emerged as a model organism especially for limb regeneration. Telomeres and the telomerase enzyme are crucial for regeneration and protection against aging processes and degenerating diseases. Despite its relevance for regeneration, the axolotl telomerase and telomere length have not yet been investigated. Therefore, in the present paper, we reveal the sequence of the axolotl telomerase reverse transcriptase gene (*Tert*) and protein (TERT). Multiple sequence alignment (MSA) showed the known conserved RT- and TERT-specific motifs and residues found in other TERTs. In addition, we establish methods to determine the *Tert* expression (RT-PCR) and telomerase activity (Q-TRAP) of adult axolotl and blastema tissues. We found that both differentiated forelimb tissue and regenerating blastema tissue express *Tert* and show telomerase activity. Furthermore, blastema tissue appears to exhibit a higher *Tert* expression and telomerase activity. The presence of active telomerase in adult somatic cells is a decisive difference to somatic cells of non-regenerating vertebrates, such as humans. These findings indicate that telomere biology may play a key role in the regenerative abilities of cells.

## 1. Introduction

A healthy organism manages to equilibrate degenerative and regenerative processes. Aging usually causes the decline of regenerative potential as we diagnose soaring degenerative diseases (e.g., chronic internal or neurodegenerative diseases) in the elderly population [1]. Furthermore, the ability to repair acute tissue injury is limited even at young age and is declined during aging. Understanding the mechanisms of the existing regenerative abilities, along with strategies to activate and enhance this potential, would be a great advantage for patients who suffer from degenerative diseases or are affected by serious injuries, such as amputations or burns. Additionally, these chronic diseases and acute injuries have a significant economic impact on our healthcare and general systems, as they produce high costs (e.g., treatment expenses and duration, the loss of working ability) [2]. Against the background of demographic changes and the aging population, the need to discover new therapeutic strategies facing degeneration and tissue damage is urgently required. The human organism’s regenerative potential is strongly limited. In response to acute injury, some tissues, such as bone [3,4], muscle [3,5] and epithelial tissues [6], can be repaired, provided that the defect does not exceed a critical size. Most human regenerative strategies are based upon the replacement of lost cells by means of adult stem cell proliferation. However, humans do not have the ability to regenerate complex structures, such as limbs [7].

In contrast, there are some organisms that can regenerate complex body parts during lifespan [8]. The Mexican axolotl (*Ambystoma mexicanum*) belongs to the salamander species and evolved to a model organism for regenerative biology [9]. Axolotls are neotenic, meaning they are permanently aquatic and do not undergo metamorphosis. This life cycle distinguishes the axolotl from other salamanders and, furthermore, differences regarding the regenerative potential and mechanisms have been reported [10].

The axolotl is capable of rebuilding its entire limbs and tail, the upper and lower jaw, the brain, spinal cord and myocardial muscle after amputation or severe damage. Deep skin wounds are described as healing without the appearance of scars [11]. Limb regeneration can be subclassified into three overlapping phases. Following amputation and rapid hemostasis, epidermal cells migrate across the amputation surface and close the wound within hours [12]. During the next days, blastema cells accumulate beneath the new built wound epidermis. These cells originate from stem cells or derive from dedifferentiation of adjacent cells [10,13]. The blastema cells re-enter the cell cycle and become highly proliferative, so that the blastema grows [14]. Once the blastema has reached a critical size (“palette” stage), the redifferentiation of the various tissues takes place and a structurally and functionally perfect limb is rebuilt [15,16].

Each chromosome’s end is capped by a DNA–protein complex known as the telomere. The denomination “telomere” derives from the greek “télos" (end) and “méros” (part) and was provided by Hermann Müller in 1938 [17]. In vertebrates, the telomeres consist of thousands of TTAGGG-repeats that build a protective cap in collaboration with associated proteins to ensure genomic stability and integrity [18,19]. During genome duplication, telomeres shorten by about 50–200 bp, due to the incomplete replication of chromosome ends (end replication problem). This progressive loss of telomeric DNA limits the number of cell cycle divisions [20]. The cell loses its proliferative potential and achieves cellular senescence [21].

Telomerase is a reverse transcriptase (RT) catalyzing the elongation of telomeres [22]. Its catalytic core consists of the protein subunit (telomerase reverse transcriptase, TERT) and the RNA subunit (telomerase RNA, TR). The TR provides the template for telomeric repeats synthesis and contains motifs essential for telomerase activity. The TERT-protein is composed of three domains: the N-terminal part, the reverse-transcriptase domain (RT-domain) and the C-terminal extension (CTE). In addition, different accessory proteins adjust telomerase’s localization, regulation and function [23]. Telomerase stabilizes telomere length by adding telomeric repeats to its ends and thus protects cells against senescence. In humans, active telomerase can only be found during embryonic development [24] in an adult’s highly proliferative cells (e.g., germ cells [24], stem cells and progenitor cells [25], and activated lymphocytes [26]) and in many tumor cells [27]. Adult somatic tissues reveal no detectable telomerase activity [28]. In several animal investigations, a correlation between remarkable regenerative capacities and high telomerase activity could be shown without noticing many cancer diseases or even a higher carcinogenic risk at all [29,30]. These findings emphasize the growing interest in developing and exploring new therapeutic strategies to improve regeneration by recovering stable telomeres [31].

Telomeres and the telomerase enzyme are important elements for regeneration and protection against premature aging and degenerating diseases. One of the best-known molecular aging mechanisms is the progressive telomere attrition and proliferative senescence when reaching a critical telomere length. Highly proliferative cells are crucial for regeneration, so telomere biology is supposed to play a significant role in regenerative processes [31]. Related to the axolotl’s excellent regenerative abilities, we also suspect this decisive role of telomere biology. 

This study aims to provide the first insights into the axolotl’s telomere biology. We identify and characterize the axolotl *Tert* gene (Amex *Tert*) for the first time. Subsequently, the methods used to measure *Tert* mRNA expression and telomerase activity are evolved and established. This information is useful for further studies concerning axolotl’s telomere biology and for the discovery of new telomere-based regeneration strategies for humans.

## 2. Materials and Methods

### 2.1. Animal Husbandry and Handling

For all the experiments, the axolotl’s adult limb tissue and blastema limb tissue were used. The 15 animals overall were bred and kept at the Ambystoma Mexicanum Bioregeneration Center of the Department of Plastic, Aesthetic, Hand and Reconstructive Surgery, Hanover Medical School. The experiments were approved by the Lower Saxony State Office for Consumer Protection and Food Safety under approval number: AZ 33.14-42502-04-13/1212 and in accordance with the German Animal Welfare Care. Adult axolotls (age: 1–5 years; length: 15–25 cm) were maintained in small groups (3–5 animals) in aquariums with fresh, warm tap water (max. 20 °C) without artificial illumination. They were protected against direct sun exposure and were fed 2 times a week with 4–5 pellets, “Axobalance” (Aquaterratec, Broeckel, Germany). Each tank was equipped with ground substrate, artificial plants, hiding places and a filter. 

Animals were anesthetized in 0.1% Tricaine (MS-222^®^) in fresh cold tap water until deep anesthesia was reached (proofed by expiration of the ventral reflex). One forelimb per animal was amputated distal to the elbow with sterile sharp scissors. Afterwards, the animals were transferred into boxes filled with fresh cold tap water. After awakening, the animals were left for one night in quarantine and then transferred back to their tanks. When the regenerating blastema reached the palette stage [15,16], a second amputation was carried out under anesthesia, as described above. Each amputated limb was cut into three slices, directly frozen into liquid nitrogen and stored at −80 °C for the following procedures. After successful regeneration, the animals were examined by a veterinarian and re-homed to private keepers.

### 2.2. Cloning of Amex Tert Gene Fragments

Contigs with sequence identity to human TERT were searched in the Ambystoma Mexicanum V4.0 (contigs) database, via its website, Sal-Site^TM^ [32]. Five Contigs (contig282000, contig 346319, FUQAVB301DCODH, GFW9XCP01AHQ6K, and GHXAJEM01EV71Q) were assembled and used for Primer derivation. The primers’ sequences were (5′-AAATGGTTTGCGCCCGATAGTC-3′ and 5′-ATAAAGGCATGGTAGCTAAGCCACTG-3′). Axolotl embryonic cDNA isolated for other projects was obtained from laboratory stock for PCR establishment. PCR was performed utilizing the Advantage^®^ 2 PCR Kit (Takara Bio Inc., Kusatsu, Japan). The amplificate was purified by Nucleo Spin^®^ PCR Clean up Kit (Macherey Nagel) and cloned into pUC118 vector [33] using the Mighty Cloning Reagent Set (Blunt end) (Takara Bio Inc., Kusatsu, Japan) according to the manufacturer’s protocol. The cloned DNA was sequenced by Sanger sequencing using an M13 primer (GATC Services, Eurofins Genomics, Ebersberg, Germany). A BLASTn [34] search in the Nucleotide Collection (nr/nt) database exhibited a high concordance with other TERT nucleotide sequences, so this cloned fragment was supposed to be part of Amex *Tert*.

### 2.3. Completion and Sequence Analysis of the Amex TERT Amino Acid Sequence

The predicted Amex TERT fragment was searched in the Transcriptome Shotgun Assembly (TSA) database using BLASTn and could be verified as part of the transcript Ambymex_c1080993_g3_i1 (GenBank Accession number GFBM010789021). The nucleotide sequence was translated into an amino acid sequence [35]. Multiple sequence alignments were performed with the Clustal Omega program (European Bioinformatic Institute, EMBL-EBI) [36]. A phylogenetic tree was constructed by the neighbor-joining method using the FigTree v1.4.3 software. The GenBank accession numbers for multiple sequence alignment and phylogeny are as follows: hTERT, *Homo sapiens* TERT (accession: NP_937983.2); mTERT, *Mus musculus* TERT (accession: AAC09323.1); TmtTERT, *Terrapene mexicana triunguis* TERT (accession: XP_024050859.1); PvTERT, *Pogona vitticeps* TERT (accession: XP_020652818.1); xTERT, *Xenopus laevis* TERT (accession: AAI70415.1); ZfTERT, *Danio rerio* TERT (accession: NP_001077335.1); OlaTERT, *Oryzias latipes* TERT (accession: NP_001098286.1); TtTERT, *Tetrahymena thermophila* TERT (accession: AAC39135.1); AtTERT, *Arabidopsis thaliana* TERT (accession: AAD54777.1); Sc_Est2p, *Saccharomyces cerevisiae* TERT (Accession: PJP08600.1); and *HIV-1* RT (accession: AAB24839.1).

### 2.4. Analysis of Amex Tert mRNA Expression

Total RNA was prepared from axolotl limbs and limb blastemas by the ArrayPure^TM^ Nano-scale RNA Purification Kit (Epicentre^®^, Madison, WI, USA) with little adaptions (quadruple amount lysis solution and higher concentrated Proteinase K (50 μg/μL), double amount MCP reagent and isopropranol). A total of 1 μg of total RNA was used for reverse transcription by iScript^TM^ cDNA Synthesis Kit (Bio-Rad, Hercules, CA, USA) according to the manufacturer’s protocol. For real-time PCR analysis, the following primer pair was established and validated: TeloExp For (5′-CCTCAAGCGTTTGTGTGTCA) and TeloExp Rev (5′-TGGCTGCTCCATAAGCCTAA). The gene ornithine decarboxylase was used as positive control and housekeeping gene [37] for 2^−ΔΔCt^ method. Therefore, the primer pair of axolotl ornithine decarboxylase (sequence listed in Table S1) was included in the PCR reaction. Real-time PCR was performed with an iCycler^TM^ using the SsoFast^TM^ EvaGreen^®^ Supermix (Bio-Rad, Hercules, CA, USA). PCR conditions: 1 s at 95 °C; 35 cycles: 10 s at 95 °C and 30 s at 56.1 °C. Afterwards, a melting curve was generated to analyze the specificity of PCR products. Data analysis was performed by 2^−ΔΔCt^ method [38].

### 2.5. Analysis of Telomerase Activity

Frozen tissue samples of axolotl limbs and limb blastemas were dispersed in a mortar and transferred into 200 μL of NP-40 lysis buffer. All samples were incubated for 30 min on ice followed by centrifugation at 16.000× *g* for 20 min at 4 °C. The clean supernatant was collected in a fresh Eppendorf tube and the total protein concentration was quantified using the Bradford assay. Telomerase activity was detected by the real-time quantitative telomerase repeat amplification protocol (Q-TRAP), according to Herbert et al. [39]. Telomerase positive samples (human MCF-7 breast carcinoma cells [40]) were used as positive control and for generation of a standard curve. For this purpose, a 1:5 dilution series was created. PCR amplification was performed as indicated by the authors [39]. With each PCR run, three negative controls were tested (lysis buffer only, RNase-treated sample, heat-inactivated sample) and each sample was analyzed at least in duplicates. After PCR, the real-time data was collected, the standard curve was determined and converted into Relative Telomerase Activity (RTA) units performing the following calculation: RTA of an unknown sample = 10^[(Ct sample–Y int)/slope]^. The standard curve obtained was: y = −6.192x + 16.336.

## 3. Results 

### 3.1. Identification and Characterization of Amex TERT

**Figure 1:**
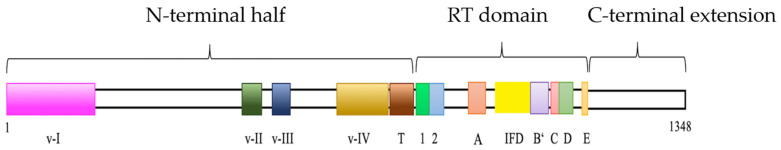
Schematic structure of the Amex TERT protein. *Tert* cDNA contains a 4848 bp nucleotide sequence encoding a 1348 amino acid sequence. Four regions conserved among vertebrates (v-I to v-IV) and motif T are located in the N-terminal half of TERT. The middle of the protein contains the seven RT motifs 1, 2, A, B’, C, D and E and the IFD. The C-terminal end is formed by the CTE. All highlighted regions were examined using multiple sequence alignment (MSA) and are shown in the Supplementary Materials (Figure S2).

Using the database “*Ambystoma mexicanum* V4.0 (contigs)”, six contigs with concordance to hTERT could be located. Based on the resulting sequence, primers were generated and PCRs amplifying axolotl cDNA performed. The PCR product was cloned and sequenced. A BLASTn search confirmed a high consensus with other TERTs, so the sequence was supposed to be part of the axolotl TERT nucleotide sequence. In the database “transcriptome shotgun assembly”, the sequence could be found as part of the transcript Ambymex_c1080993_g3_i1 (GenBank Accession number GFBM010789021) [41] with 99.41% identity. This transcript was confirmed as an axolotl TERT nucleotide sequence (as is shown below) and collected in GenBank (GenBank Accession number MK702005) [42]. Therefore, we designated this gene as Amex *Tert*. The 4848 bp nucleotide sequence encoded a protein sequence composed of 1348 amino acids (aa) (GenBank Accession number GQJ02387) [43]. Figure 1 shows the schematic structure of the Amex TERT protein. The entire TERT amino acid sequence shows 53.34% identity to *Terrapene mexicana triunguis* TERT (TmtTERT); 52.17% to *Xenopus laevis* TERT (xTERT); 46.13% to *Mus musculus* TERT (mTERT); 45.99% to *Homo sapiens* TERT (hTERT) and *Pogona vitticeps* TERT; 35.37% to *Oryzias latipes* TERT (OlaTERT); 34.71 to *Danio rerio* TERT (ZfTERT); 22.35% to *Arabidopsis thaliana* TERT (AtTERT); 19.62% to *Tetrahymena thermophila* TERT (TtTERT); and 19.02% to *Saccharomyces cerevisiae* Est2p (Sc_Est2p).

Multiple sequence alignment (MSA) revealed that TERT contains several RT- and telomerase-specific motifs. At its C-terminal half, TERT possesses the telomerase-specific motif T [44] (aa 763 to 810) followed by the 7 RT motifs 1, 2, A, B’, C, D and E [45] (aa 818 to 1151). The RT motifs are found in diverse groups of RT, but there are short telomerase-specific signatures within these RT motifs that distinguish TERT from other RT [44], which can also be found in Amex TERT. They include a conserved arginine (R) in motif 1 and a conserved phenylalanine (F) behind two aspartic acids (D) in motif C and tryptophan (W)-x-glycine (G)-x-leucine (L) in motif E. Amex TERT also contains amino acid residues essential for RT activity [46]. Between motif A and B’, the TERT-specific domain called “insertion in fingers domain” (IFD) is shown [47]. The domain behind motif E to the C-terminal end of the sequence is called “C-terminal extension”. This area does not reveal many similarities to conventional RT, but has a high concordance to other vertebrate TERT [23,48]. The analysis of the N-terminal half of Amex TERT revealed four known regions conserved among vertebrate TERTs (v-I to v-IV) [49].

**Figure 2:**
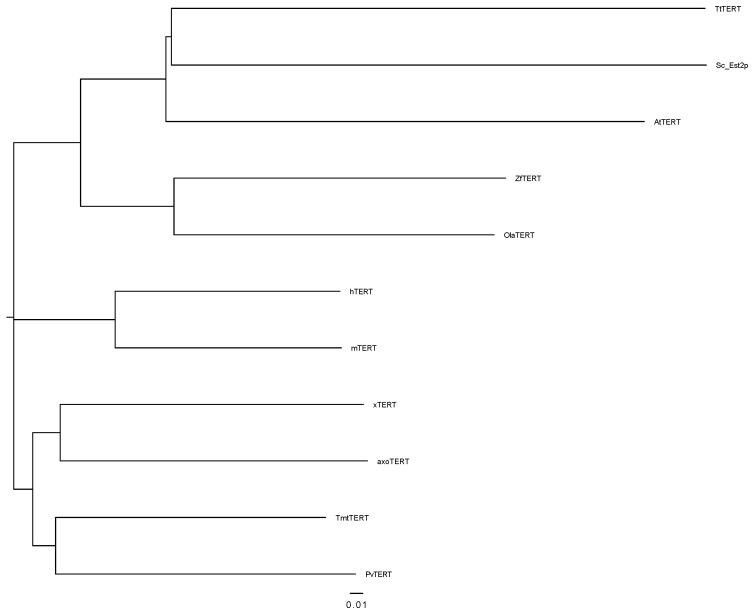
Based on the MSA (Figure S2), a phylogenetic tree of TERT proteins was created according to the neighbor-joining method. Scale bar indicates 0.1 changes per amino acid.

The MSA of complete TERT protein and all the mentioned RT- and telomerase-specific motifs is presented in the Supplementary Materials (Figures S1 and S2). Based on the multiple sequence alignment of different TERT proteins, a phylogenetic tree is constructed according to the neighbor-joining method (Figure 2). As expected, Amex TERT shows a higher sequence identity and similarity to those of vertebrates than to plant, yeast and ciliates. 

### 3.2. Expression Analysis of Amex Tert mRNA by RT-PCR in Somatic and Blastema Tissues

We established a method to determine the *Tert* mRNA expression by RT-PCR. Therefore, the primer TeloExp was created and validated. The gene encoding ornithine decarboxylase was used as a positive control and reference gene for relative quantification. RT-PCR revealed that Amex *Tert* mRNA was expressed in both adult limbs and limb blastemas. All samples exhibit *Tert* expression, whereas a higher expression could be detected in blastemas (Table 1). Raw PCR data are shown in the Supplementary Materials (Table S1). 

### 3.3. Active Telomerase Is Detectable in Axolotl Adult Limbs and Limb Blastemas

**Figure 3:**
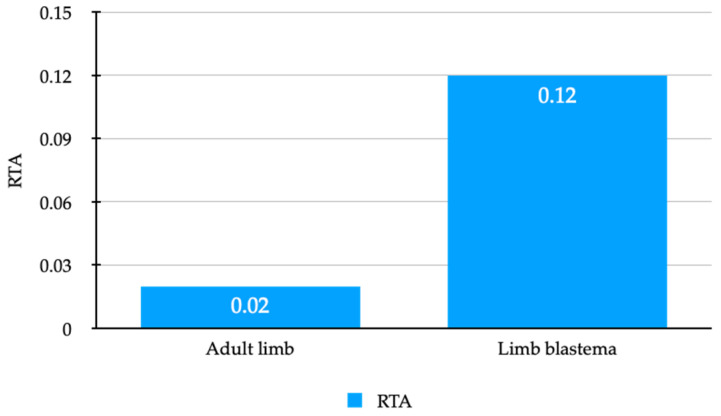
Difference in RTA for adult limbs and limb blastemas.

Simultaneous to the expression analysis, we adapted Q-TRAP [39] to axolotl tissue for examining a possible correlation between Amex *Tert* expression levels and telomerase activity. Protein extracts of adult limbs and blastema limb tissues were used as samples and MCF-7 protein extracts served as positive controls and references (the standard curve is shown in the Supplementary Materials (Figure S3)). All negative controls (heat-inactivated and RNase-treated) remained negative. In contrast to most non-regenerating vertebrates, axolotl somatic limb tissue exhibits detectable telomerase activity. According to the findings for *Tert* expression, the blastemas reveal a higher telomerase activity, so telomerase activity seems to be upregulated during limb regeneration (Table 2, Figure 3). 

## 4. Discussion

Although the importance of telomeres and telomerase for mammalian aging, cancer and regeneration and the axolotl’s outstanding regenerative abilities were extensively discussed, to date, there is a lack of research investigating its telomere biology. In this study, we identified and characterized the axolotl TERT gene. The existence of the 7 RT motifs (1, 2, A, B’, C, D, E) and telomerase specific motifs (motif T, IFD, short TERT-specific signatures in RT motifs, vertebrate-specific domains in the amino-terminal half) confirmed the identification of Amex TERT. The nucleotide and amino acid sequence can be found in GenBank^®^ under accession MK702005 [42] and QGJ02387 [43]. The sequence of Amex *Tert* allowed for the examination of *Tert* mRNA expression by real-time PCR. 

Real-time PCR and TRAP assay demonstrated that limb blastemas, as well as differentiated adult limbs, express TERT mRNA and show telomerase activity. Both appear to be at higher levels in blastemas. This concordant relationship between TERT expression and telomerase activity was already found in other species (e.g., human [44], *Xenopus laevis* [49]). The presence of telomerase activity in somatic tissues of axolotl is a clear contrast to mammalian tissues, in which telomerase activity can be found in embryonic tissues, germline cells and stem cells, but is not detectable in most adult somatic tissues [24]. Other organisms (e.g., nonvertebrate [50], fish [51,52], some amphibian [49] and reptiles [18]) reveal TERT expression and telomerase activity, even in somatic tissues. At the same time, these animals exhibit notably regenerative abilities and hardly any signs of senescence during lifespan (age-related diseases, loss of reproductive abilities). Therefore, it is reasonable that the presence of TERT expression and telomerase activity is associated with high regenerative capabilities. The higher levels of TERT expression and telomerase activity in limb blastemas indicate that during regeneration, TERT expression is upregulated. Similar observations were made in axolotl tails [53], similar to other regenerative species (e.g., *Aeolosoma viride* [50]), *Danio rerio* [54]). Regeneration in the axolotl is performed by the dedifferentiation of somatic cells. Our results show that during the process of dedifferentiation, the upregulation of *Tert* mRNA expression occurs. The underlying mechanisms have to be investigated in the future. In the next step of regeneration, the dedifferentiated blastema cells start to proliferate. Because of active telomerase, the telomeres persist and cellular senescence is avoided. The length of the axolotl’s telomeres has not yet been investigated, but the high variable telomere lengths of other highly regenerative organisms suggest that an active telomerase is crucial for regeneration, rather than the length of the telomeres.

The axolotl differs from other mentioned species with regenerative abilities. It is one of the few vertebrates able to regenerate complex structures (e.g., limbs) throughout its lifespan. In contrast, *Xenopus laevis* regenerates whole limbs prior to metamorphosis, but progressively loses this ability, simultaneously with the initiation of metamorphosis [55]. Similar observations were made in mammals: in the early development stage, structures, such as the heart muscle, spinal cord and digits, can be rebuilt. During further development, these abilities certainly expire [31]. Congruent to these findings, in early development stages, higher levels of telomerase activity are detectable in human cells, in which the telomerase activity is suppressed later on. A possible explanation for axolotl’s lifelong regenerative abilities may be based on the neoteny (meaning they become sexually mature without metamorphosis) and preservation of some embryonic-like characteristics [56]. To what extent telomere biology in the axolotl is associated with neoteny has to be examined in further studies. In mammals, the absence of telomerase activity in somatic cells is explained by the need for efficient protection against tumor formation. Without telomerase activity, the telomeres shorten with every cell cycle and proliferation is limited [20,57]. This protection against tumor formation seems to be so important that these organisms accept deficient regenerative capabilities.

Humans possess very limited regenerative abilities that decline with age. Most tissues respond to trauma or diseases with inoperable scar formation. Complex structures cannot be replaced at all. During lifespan, age-related diseases and changes appear [7]. Nowadays, therapeutic strategies that counteract these defects in regenerative capacities are lacking. For developing an opportunity to induce regeneration in adult human tissues, it is useful to explore the already existing mechanisms that enable perfect regeneration. The axolotl uses a mechanism of dedifferentiation, which returns adult somatic cells to a more youthful, undifferentiated status. Interestingly, this type of regeneration can be observed in mammals, too. Mice are able to regenerate their digits by building a blastema at all stages of development (including adults). Human fingertip regeneration parallels digit tip regeneration in mice [58,59]. This fact strengthens the hypothesis that humans own the essential requirements for perfect regeneration and it raises the relevance of the axolotl as a model organism for regenerative biology. In this study, we focused on telomere biology’s role in regenerative processes in the axolotl, and demonstrated the importance of TERT expression and telomerase activity for limb regeneration. Our findings, combined with the known relationship between short telomeres and age-related diseases, point out the possible therapeutic potential of active telomerase. It was already shown that reactivation of telomerase in mice prohibits age-related diseases [60]. The major concern and thereby main obstacle to use telomerase activation as a therapeutic strategy is the possible increase in cancer risk. Over 80% of malignant tumors exhibit high telomerase activity. Studies from telomerase-deficient mice suggest that telomere shortening leads to chromosomal instability and cell transformation. A following telomerase activation allows for cell immortalization and cancer progression. However, telomerase activation in cells with stable chromosomes can prevent telomere shortening and thus tumor initiation. The telomerase itself is not an oncogenetic factor, but enables the unlimited proliferation of already transformed cells [28]. 

## 5. Conclusions

To develop a therapeutic strategy based on the axolotl’s regeneration mechanisms, further investigation is required. In this study, we identified and characterized the axolotl TERT gene (Amex *Tert*). To date, this is the first report of the axolotl *Tert* gene and TERT protein. Additionally, we established methods to determine *Tert* mRNA expression and telomerase activity in axolotl limb and blastema tissues. This enables further studies with an increased number of animals to be used to examine telomerase expression, regulation and function during regeneration in different tissues and organs. Additionally, attention should be paid to the telomerase’s RNA subunit (TR), which is essential for telomerase activity. The TR has to be identified and examined to extensively understand the reconstitution of telomerase activity. Furthermore, studies concerning telomere length in axolotl cells are required to elucidate axolotl’s telomere biology.

## Figures and Tables

**Table 1:** Difference in Amex *Tert* gene expression for adult limbs and limb blastemas. The mRNA levels were determined by real-time RT-PCR in adult limbs and limb blastemas, and related to the reference gene ornithine decarboxylase in both tissues.

	*Amex TERT* Average C_t_	*Ornithine* Average C_t_	△C_t_ (C_t Amex *TERT*_ − C_t *Ornithin*_)	△△C_t_ (△C_t Blastema_ − △C_t Limb_)	Fold Difference (2^−ΔΔCt^)
Adult limb (n = 7)	31.19 ± 1.81	31 ± 1.67	0.19 ± 2.46	0	1
Limb blastema (n = 6)	30.42 ± 1.22	30.93 ± 1.53	−0.51 ± 1.96	−0.7 ± 1.96	1.62 (0.42–6.32)

**Table 2:** Difference in RTA for adult limbs and limb blastemas. Telomerase activity was measured quantitatively in adult limbs and limb blastemas with Q-TRAP assay using 1 μg of protein extract.

	Average C_t_	RTA
Adult limb (n = 4)	26.6 ± 2.23	0.02 (0.01–0.05)
Limb blastema (n = 3)	22.02 ± 1.69	0.12 (0.06–0.23)

## Data Availability

The Amex TERT nucleotide and amino acid sequences were deposited in NCBI GenBank under the accession number MK702005 and GQJ02387.

## Supplementary Materials

The following supporting information can be downloaded at: https://www.mdpi.com/article/10.3390/genes13020373/s1; Table S1: RT-PCR raw data using primer TeloExp and adult limb mRNA, respectively, limb blastema mRNA; Table S2: Primer sequences; Figure S1: Multiple sequence alignment (complete TERT protein); Figure S2: Comparison of different TERT motifs of representative species using MSA; Figure S3: Determination of the standard curve and linear relationship for Q-TRAP.
