# Supplementary material for: An Identification and Characterization of the Axolotl (Ambystoma mexicanum, Amex) Telomerase Reverse Transcriptase (Amex TERT)
Source: Genes (Basel). 2022 Feb 18;13(2):373. doi: 10.3390/genes13020373 (PMC8924892; doi:10.3390/genes13020373)
Supplement: Supplementary file 1 [file genes-13-00373-s001.zip › genes-1577429 - supplementary/Figure S1.pdf]

```
AmexTERT    ----MQRGNDFAGVLRLLRSVYPQ--------VCTLIQFM---------PTFPV----VH   35
hTERT       ----MPRAPRCRAVRSLLRSHYRE--------VLPLATFV---------RRLG------P   33
mTERT       ----MTRAPRCPAVRSLLRSRYRE--------VWPLATFV---------RRLG------P   33
TmtTERT     ----MAGSGPFRAVLAMLEGCYAE--------VLGLEQFV---------QRLREAAACGP   39
PvTERT      ----MGRPRRWRAVQGLLRSCYAE--------VLPLEAFV---------ERLQGQAQAEG   39
xTERT       ----MPLRTGGATLLSILQRLYGQ--------VLGIVEYT---------DTLQVP---GG   36
ZfTERT      MSGQYSTDGGFRPVLEILRSLYPV--------VQTLEEFT---------DGLQFP---DG   40
OlaTERT     -----MTSGDLSSVLNILRSLYKR--------TRTLEEFA---------DGVVFR---EG   35
TtTERT      ------------------------------------------------------------   0
AtTERT      ----MPRKPRHRVP-EILWRLFGNRARNLNDAIVDLIPNRNIQPEQCRCRGQGCLGCSSD   55
Sc_Est2p    ------------------------------------------------------------   0

AmexTERT    GSATIQRSDDGEHYRTFVSQLLVCIPGEAWPPPHSAKIQQLSSQREVVARVIQRICEK-Q   94
hTERT       QGWRLVQRGDPAAFRALVAQCLVCVPWDARPPPAAPSFRQVSCLKELVARVLQRLCER-G   92
mTERT       EGRRLVQPGDPKIYRTLVAQCLVCMHWGSQPPPADLSFHQVSSLKELVARVVQRLCER-N   92
TmtTERT     GEAELLQRGDSELYQTFVSQCVVCVPRGARAIPQPLSFQQLSSESEVVIRVIQRLCEK-K   98
PvTERT      PLVPLVQEGDPACYRHLVERCLVGRAPGCKALPSRVVFQQVSSLNDVVARVIQRICEK-K   98
xTERT       IKVPVLLEGDSEKFRSFVAELVVCIPRGTKPLPSPVSFLQLSTQREVVARVIQRICEK-K   95
ZfTERT      RKPVLLEETDGARFKKLLSGLIVCAYTPPQ-LR---VPAQLSTLPEVLAFTLNHIKRK-K   95
OlaTERT     RRAALLQPSDTHSFKSFVRGVFVCSDEELQDVP---SCNQTCTFPELLAFILNSLKRK-R   91
TtTERT      ----------------------------------------------------MQKINNINN   9
AtTERT      KPAFLLRSDDPIHYRKLLHRCFVVLHEQTPPL-LDFSPTSWWSQREIVERIIEMMQSGCD   114
Sc_Est2p    ------------------------------------------------------------   0

AmexTERT    RSNVLAFGFALVDEN---SPM-PLTFAPNICNFLPNPTTATISTSVLWGTLLSRIGDDIM   150
hTERT       AKNVLAFGFALLDGA---RGGPPEAFTTSVRSYLPNTVTDALRGSGAWGLLLRRVGDDVL   149
mTERT       ERNVLAFGFELLNEA---RGGPPMAFTSSVRSYLPNTVIETLRVSGAWMLLLSRVGDDLL   149
TmtTERT     KKNVLAFGYTLLDEN---SSYSQVMSTSNICSYLPNTATETLRMSVLWETLLSRIGDDVM   155
PvTERT      KKNLLASGYSLLNEN---NYQ--LSCMPNIYTYLPNNTTETIRQSILWEIILSRVGDDVM   153
xTERT       KKNVLAFGYGLVDEK---NSL-NIRLTPNICNYFPNPTTTTISTSILWETLLTRVGDDVM   151
ZfTERT      LRNVLGFGYQCSDVT---TSSDPFRFHGDV-----SQTAASISTSEVWKRINQRLGTEVT   147
OlaTERT     RRNVLAHGYNFLGVAQEDRDADHFRFQGDL-----SQSAAYIHSSDLWKKVTARLGTDVT   146
TtTERT      NKQMLTRKEDLLTVL---KQISALKYVS-----------------NLYEFLLAT------   43
AtTERT      CQNVICARYDKYDQ---------------------SSPILELLTSSSWEFLLKRVGHDVM   153
Sc_Est2p    ------------------------------------------------------------   0

AmexTERT    MYLLEHCSLFMLVPPTCCYQICGLPVYKLLN-NVCRSPAWTRQIGSRQRGNILLMYIQKK   209
hTERT       VHLLARCALFVLVAPSCAYQVCGPPLYQLGAATQARPPPHASG----PRRRLGCE-----   200
mTERT       VYLLAHCALYLLVPPSCAYQVCGSPLYQICATTDIWPSVSASYR---PTRPVGRNFTNLR   206
TmtTERT     MYLLEHCAIFMLVPPSCSYQICGQPVYELTSSNAAPSAVFVKQRYSKSRHNILFDYVRKK   215
PvTERT      MYLLEHCALFMLVPPSCCYQMCGQPIYELALRNATLSSAFFRQRYPGPTRSALSGYLQKR   213
xTERT       MYWLEQCSIFVFVPPRCCYQITGQPIYTLPSDDV-----FLFQSQSFTQSNVLLRYIKRN   206
ZfTERT      RYLLQDCAVFTTVPPSCVLQVCGEPVYDLLMPR-SWSGFFLSNSDNERISGAMRKFPAVQ   206
OlaTERT     RYLLGSCSVFVLAPPSCIFQICGVPAYDRVSMTTASSGFLLRPPSRKHKS----------   196
TtTERT      ------EK----------------------------------------------------   45
AtTERT      VYLLQQTSIFLPLLGKKHQQVSGPPLCIKHKRTLSVH-------ENKRKR----------   196
Sc_Est2p    -------------------------------------------------MKILFEFIQDK   11

AmexTERT    --FHQHKRYLEKATFWKAKRRLQANSSVFSVQRSSQTQHCT-GNCQNGKTQLVLVEPHPS   266
hTERT       ----R---------AW-----------NHSVREAG------------VPLGLPAPGAR--   222
mTERT       -FLQQ---------IK-----------SSSRQEAP------------KPLALPSRGTK--   231
TmtTERT     --FQLSKTN-----RWKWKPRSEINILNTKSQRNNKTQGSV-PDYEHSATAKGAVGQN--   265
PvTERT      --LKSCGKF---------LATANRKKGNSRSQKSRKIQKGLECDCRLSPMGYEVHGLKPA   262
xTERT       -VFHLRKKYLKPKHSM------TSRMLTW---RRNK-----------SPSGLLIRS----   241
ZfTERT      KTVAISKK----------------------RTRDN--------EKYIS------------   224
OlaTERT     --FQVGKK----------------------RRSANLTKRGSVGDVEES------------   220
TtTERT      ------------------------------------------------------------   45
AtTERT      ------DDNVQPPTKRQWLSSAV--------------DD---CPKDDSATITPIVGEDV-   232
Sc_Est2p    LDID--------------------------LQTNST------------------------   21

AmexTERT    PGKSHAMQQHVTDNHL--------KRKST--LRPATTHENEDEAGVPAKRMRRDFIGTQA   316
hTERT       ---RRG---GSASRSLPLPKRPRRGAAPEPERTPVG----------------------QG   254
mTERT       ---RHL---SLTSTSVPSAKKARCYPVPRVEEGPHR----------------------QV   263
TmtTERT     -SKTNKQV-RMATGHLENRRGPNLYPVAT--TTPSKRKLDRDQFEVTAKRMKINLREEED   321
PvTERT      KSKPFAGM-SICRESV--QRSDQC--TSL--TAPLLKRKWTGQCGTSAKRVKIMQTEEEL   315
xTERT       --KT--SM-AVTT-EI----------------HSKRKLCSKDICVIPDKKRRDNLDKDDT   279
ZfTERT      -VKRRRVKETVNNNN--GNYRSLCFAI-------SKKRAIDNEENISLKRRRMEETDQVA   274
OlaTERT     -RKRRRVESGVSA----------------------RKRKREPEEEESRERRRGVHHE---   254
TtTERT      ---------IVQTSEL------------------------DTQFQEFLTTTIIA--SEQN   70
AtTERT      --------------------------------DQHREKKTTKRSRIYLKRRRKQRKVNFK   260
Sc_Est2p    ------------------------------------------------------------   21
```

```
AmexTERT        Q----SFCSLVNKSVPN-LKTICDR------------CGWKSMVDLATPTEIQAVKPDVG  359
hTERT           SWA-----------HPG-RT---RG---------PSDRGFCV-VSPARPA-------E--  280
mTERT           LP----------------------T---------PSGKSW-V-PSPARSP-------EVP  283
TmtTERT         AW---NLGSNVDKNKFT-LDIIYDTQ-SLSFVESFSVNNFIKIKSISQENEGTAETPDAP  376
PvTERT          EKEMRNLVPTKSKNIVM-LDDGGDGSTSKRCARLCTEDGLIVVKSVPQSPECGAQMSNVP  374
xTERT           V------------DHFD-LP----------------MCGSVSYLSNMY--------PKTN  302
ZfTERT          KIR--------NENHES-------------------------------------------  283
OlaTERT         --E--------RRQHEA------------------------V------------------  262
TtTERT          L--VENYKQKYNQPNFSQLT----------------------------------------  88
AtTERT          K---------VDCNAPC-IT------------------------------------PSTN  274
Sc_Est2p        ------YKENLKCGHFNGLDEI-------------------LTTCFALPNSRKIALPCLP  56

AmexTERT        SRLQNKVD---IPVCEDSLS-PASPFEANR-----RET-----HVVEENS-------EKF  398
hTERT           ----------------EATS-LE------------GAL----------------------  289
mTERT           T-------------AEKDLS-SK------------GKV----------------------  295
TmtTERT         SLVQEVQDSKEKSVTDDENS-LRLRVQCNKLYKSSTET-K-------TNS-------SR-  419
PvTERT          YAVPDNQE----------------SFLCDKILTLDNES-E---TLCELNS-------RR-  406
xTERT           VQVT--------GLITSGYK-KTKTFQCQKPVSCEQKK-T-----------------TAF  335
ZfTERT          --------------QSFAIS---------------KKR-A---RDNEENI-------SL-  302
OlaTERT         -------------LDESTLS---------------GKS-G------ENDA-------A--  278
TtTERT          ----------IKQVIDDSIILLGNKQNY--VQQIGTTTIGFYVEYENINLSRQTLYSSNF  136
AtTERT          GKVSTGN---------D-----------------------------EMNL-------HI-  288
Sc_Est2p        GDLSHKAV------IDHCIIYLLTGELYNNVLTFGYKI-A---RNEDVNN-------SLF  99

AmexTERT        GNVLHLPKI--------RNNVTT--ECITLQEKAYSVKCCKDQLFREEIKEA---KNNW-  444
hTERT           -S-----GT--------R--------------HSHPSVGRQHHAG---------------  306
mTERT           -S-----DL--------S--------------LS-GSVCCKHKPS---------------  311
TmtTERT         -----------------E---------------GVELDICKFQVGSVQMKKA---EDTFL  444
PvTERT          ESSTRQPKL--------R---------------TGDSDVCFGQPL-----------GTEM  432
xTERT           YS------------------V------------AGD---CNL-----SLKDN---VNKL-  353
ZfTERT          ----KRQRM--------E-------------EIDQVAKIRNE------------------  319
OlaTERT         -------AV--------K-------------PPPETSAAPPP------------------  292
TtTERT          RNLLNIFGEEDFKYFLIDFLVFTKVEQNG---YLQVAGVCLNQYFSVQVKQKKWYKNNFN  193
AtTERT          -------GI--------N----------------GS-------------------LTDF-  297
Sc_Est2p        CHSA---NV--------NVTLLKGAAWKMFHSLVGTYAFVDLLINYTVIQFN---GQFFT  145

AmexTERT        ----QEAKPTK---SVSK-Q------PTQILPSN----IIIERGYLLYS-NFSFR-----  480
hTERT           --------PPS---TSRPPR------PWDTPCPP----VYAETKHFLYS-SGDK------  338
mTERT           --------STS---LLSPPR------QNAF-QLR----PFIETRHFLYS-RGDGQ-----  343
TmtTERT         KYRGLKCPPTN----IAKKL------SNKISRSR----VYIGRKGLLYS-SRSFR-----  484
PvTERT          TSGGRGCKTRV---SKSVQN------SDRSSVPV----TYIERRFLLYC-HRQLK-----  473
xTERT           --------ITN---ASVPTA------QSRLSFSN----IFIDFGRTLYL-SISYK-----  386
ZfTERT          NHGSQS---------WKPAD------QRPPRPSQ----CSIRVLSMLYN-GRGM------  353
OlaTERT         LEGGPS---------WRSGA------FPPLPSSQ----CFIRTLGFLYG-GRGM------  326
TtTERT          MNGKATSNNNQ--------N--NANLSNEKKQENQYIYPEIQRSQIFYCNHMGREPGVFK  243
AtTERT          ---------------VK--------------QAK----QVKRNKNFKFG-LSETY-----  318
Sc_Est2p        QIVGNRCNEPHLPPKWAQRSSSSSATAAQIKQLT----EPVTNKQFLHKLNINSS-----  196
                                                               .
AmexTERT        ------EGFSVSFLLNTLKQCPFGGQ-----RLVENIFLSRGPFEHHPVNKPATTS-KKW  528
hTERT           ------EQLRPSFLLSSLRPSLTGAR-----RLVETIFLGSRPWMPG-------TP-RRL  379
mTERT           ------ERLNPSFLLSNLQPNLTGAR-----RLVEIIFLGSRPRTSG-------PL-CRT  384
TmtTERT         ------ECFPKSFLLNRLKGCQAGGQ-----RLVETIFLNSKMLVQKCSENTPSYN-WRK  532
PvTERT          ------ERLPESFILNRLKSFSSGGR-----RLVEVIVLDSKITKQPDSINPRSSG-KRK  521
xTERT           ------KGFSESFILNSLDSTPSGSQ-----KLVETIFLSNFLAEQNF-DQPKRDE-NCR  433
ZfTERT          ----------KNFLLNRKLKGVGGARRMQGEDLVRMIFLQS----ES-------ND-SKP  391
OlaTERT         ----------HGFCLNRKRRTAAGPRRLQGQDLVRLVFFEGLPYLNG------QE-RKP  368
TtTERT          SSFFNYSEIKKGFQFKVIQEKLQGRQ-----------FINSD------KIKPDHPQTIIK  286
AtTERT          ------SVIPPNHILKTLRPNCSDSK-----LLMNHIFGEVNVWSTTPSHG---KG-NCP  363
Sc_Est2p        ------SFFPYSK----ILPSSSSIK--KLTDLREAIFPTNLV--------------KIP  230

AmexTERT        KRLPKRYWP-----------MKCVFQELVDNHNKCPYKVLLRNNCPVRLPQVSLESNASL  577
hTERT           PRLPQRYWQ-----------MRPLFLELLGNHAQCPYGVLLKTHCPLRAAVTPAAGV---  425
mTERT           HRLSRRYWQ-----------MRPLFQQLLVNHAECQYVRLLRSHCRFRTANQQV------  427
TmtTERT         KRLPRRYWQ-----------MRDVFQKLLKNHAKCPYLVLLKKNCPVWISETNSSKTDTM  581
PvTERT          RRLPKRYRE-----------MRDLFQELLENHSKCPYLKVLRKNCPVWVSESVQKSTEGE  570
xTERT           YKLPKRYWK-----------MKPHFQELIQNHKKFPYLVYLNKHCPVRSSMACSEKRSLQ  482
ZfTERT          KKLPKRFFA-----------MVPLFSRLLRQHRKCPYRLFLQRKCAGNP-----------  429
OlaTERT         KKLPLRYFN-----------MVPVFGRLLQRHRKCRYSSVLHRMCPVVELSRA-------  410
TtTERT          KTLLKEYQSKNFSCQEERDLFLEFTEKIVQNFHNINFNYLLKKFCKLPENYQSLKSQVKQ  346
AtTERT          SGSICLYHS-----------LLKSLKNLIGKTKSSHLKMLLDKHCPVLLLQEDALKSGTT  412
Sc_Est2p        QR---LKIR-----------MNLTLQKLLKRHKRLNYVSILNSICPPLEGTVLD------  270
```

```
AmexTERT      EKSV-CG-VP--GPPHVDRGKAPNVQENTDERERSESTTLSGCKMEVAYEFSQVEAKHSS   633
hTERT         CAR-----------------------------------------EKPQGS---------   434
mTERT         -----------------------------------------------------------   427
TmtTERT       CQSVSCDGVQIDREAEGQLGKEPPIC--------LTSNGREGGYTAVPNGSCSLSKRTST   633
PvTERT        SD----------KEKDHRKGRSSSRS--------RQDTTLENCFRLAPDGLNPFPGTSE-   611
xTERT         KNRIEND----------------------------------------------------   489
ZfTERT        -----------------------------------------------------------   429
OlaTERT       -----------------------------------------------------------   410
TtTERT        -----------------------------------------------------------   346
AtTERT        SQSSRRQ--KAD--------------------------------KLPHGSSS-------   430
Sc_Est2p      -----------------------------------------------------------   270

AmexTERT      N--TLVSTVTFKEAQSSQPGKEATAQQFRDSSFMKLLRQYSSLWQVYAFARECLKTVVPK   691
hTERT         --------------------VAAPEEEDTDPRRLVQLLRQHSSPWQVYGFVRACLRRLVPP   475
mTERT         ----------------------TDALNTSPPHLMDLLRLHSSPWQVYGFLRACLCKVVSA   465
TmtTERT       DLSILSNPEKF--VLGRTENERQNAKDFCDPGLKELLRQHSSHWQVYVFVRECLDLVVPA   691
PvTERT        --DSIRKSENA--ELKRKNTEGQISPDSSSSGVMTFLKQHSTHWQVYMFVRECIERVVPA   667
xTERT         --------------------GKQLKHFTTKANLLSLLKQHSSIWQVYMFVRECLNNVVPD   529
ZfTERT        ----------------------------DVKDMESLLKSHSSPYRVYLFVRECLRHIIPH   461
OlaTERT       ----------------------------AQGELSSLIPQHCAPHRVYLFVRECLTAVVPE   442
TtTERT        ----------------IVQSENKANQQSCENLFNSLYDTEISYKQITNFLRQIIQNCVPN   390
AtTERT        -----------------SQTGKPK----C--PSVEERKLYCTNDQVVSFIWAICRYIVPE   467
Sc_Est2p      ---------------------------------LSHLSRQSPKERVLKFIIVILQKLLPQ   297

AmexTERT      ELWGSNHNKYRFFKNVKTFIS-LGKFGKFSLRELMWKMRVTDCAWLRLGK------DDHF   744
hTERT         GLWGSRHNERRFLRNTKKFIS-LGKHAKLSLQELTWKMSVRDCAWLRRSP------GVGC   528
mTERT         SLWGTRHNERRFFKNLKKFIS-LGKYGKLSLQELMWKMKVEDCHWLRSSP------GKDR   518
TmtTERT       ELWGSSHNKCRFFKNVKTFIS-LGKFARFSLQELMWKMRVNDCTWLRVIK------GDHF   744
PvTERT        ALWGSSHNKCRFYKNVKKFIS-LGKFNVFSLRELMWKMRVKNCVWLRLNK-----EQGHF   721
xTERT         IMWGSSHNKCRFFRNVKSFLFFSGKFGKISLSELMWSMRVEDCSWIRLQK------SDHF   583
ZfTERT        ELWGCQENQLHFLSNVKNFLL-LGKFERLTLVQLMWRMKVQACHWLGPKK------R---   511
OlaTERT       ELWGSDHNRLQFFSRVRGFLK-SGKFERISVAELMWKIKVMDCDWLKLRR------TAGR   495
TtTERT        QLLGKKN-FKVFLEKLYEFVQ-MKRFENQKVLDYICFMDVFDVEWFVDLK------NQKF   442
AtTERT        SLLGTTHQMRVLRKNIAWFVS-RRRNEKCTVNQFLHKVKPSDFPFFARKELCCMVNGHEL   526
Sc_Est2p      EMFGSKKNKGKIIKNLNLLLS-LPLNGYLPFDSLLKKLRLKDFRWLFISDIWFT------   350

AmexTERT      V-PASEHRFREVIFAKFLYWLMDTYVIQLLRSFFYITETMFQKNALFFYRKCIWSKLHKI   803
hTERT         V-PAAEHRLREEILAKFLHWLMSVYVVELLRSFFYVTETTFQKNRLFFYRKSVWSKLQSI   587
mTERT         V-PAAEHRLRERILATFLFWLMDTYVVQLLRSFFYITESTFQKNRLFFYRKSVWSKLQSI   577
TmtTERT       V-PALEHHYREEILAKFLYWLMDTYIVQLLRSFFYITETMFQKNMLFFYRKLIWSKLQNI   803
PvTERT        V-PSSEHRFRQDLMAKFLYWLMDSYVAELLRSFFYITETMFQKNLLFFFRKSIWSKLESI   780
xTERT         V-PASEHLLREKILAKFVFWLMDTYVIQLLKSFFYVTETMFQKHRLLFYRKSVWKKLQNI   642
ZfTERT        Q-CASEHRYREWMLGQCMGWMLSGFVVGLVRAQFYITESMGHKHTLRFYRGDVWSRLQDQ   570
OlaTERT       F-PPSELAYRTRILSQFLTWLLDGFVVGLVRACFYATESVGQKNAIRFYRQEVWSKLQDL   554
TtTERT        TQKRKYISDKRKILGDLIVFIINKIVIPVLRYNFYITEKHKEGSQIFYYRKPIWKLVSKL   502
AtTERT        Q-SESIRSTQQMLCTKWISWLFLEIVKKLVHFNFYATESQGGRLNIYYYRKRSWERLISK   585
Sc_Est2p      ---KHNFENLNQLAICFISWLFRQLIPKIIQTFFYCTEISS-TVTIVYFRHDTWNKLITP   406

AmexTERT      GLGKHF----AKVKLRSLSSRDVAALKQ---NPLISRLRFIPKPNG--LRPIVKVQNVLDVQ   856
hTERT         GIRQHL----KRVQLRELSEAEVRQHREARPALLTSRLRFIPKPDG--LRPIVNMDYVVGAR   643
mTERT         GVRQHL----ERVRLRELSQEEVRHHQDTWLAMPICRLRFIPKPNG--LRPIVNMSYSMGTR   633
TmtTERT       GMRNHF----AKVHLRALSAEEIEKVQQKKCVPMASKLRFIPKPNG--LRPIVKVNSVSGAK   859
PvTERT        GIRNHL----AKVHLRVLLKEEIKTLQQQKCVPLASKLRFIPKPNG--LRPVVKQNGVVGAE   836
xTERT         GLRKHL----EKVKLRSLSSDELENMQQWKNVPLVSRLRFIPKTNG--LRPISKISSTLSSQ   698
ZfTERT        AFRAHL----CKGQWRPLSPSQALKVPN---SAVTSRIRFIPKTSS--MRPITRLSGSR---   620
OlaTERT       AFRRHI----AKGEMEELSPAQVASLPK---GTVISQLRFIPKTDG--MRPITRVIGAD---   604
TtTERT        TIVKLEEENLEKVEEKLIPED-------SFQKYPQGKLRIIPKKGS--FRPIMTFLRKDKQK   555
AtTERT        EISKAL----DGYVLVDDAEAE-------SSRKKLSKFRFLPKANG--VRMVLDFSSSSRSQ   634
Sc_Est2p      FIVEYF----KTYLVENNVCRNHNS--YTLSNFNHSKMRIIPKKSNNEFRIIAIPCRGADE-   460

AmexTERT      LHSKEKRDRKMQYFNTQLKNLFGVLNYERTRNPNLLGAFGFGLDGISKAWRQFVLKV-LE   915
hTERT         TFRRE---KRAERLTSRVKALFSVLNYERARRPGLLGASVLGLDDIHRAWRTFVLRV-RA   699
mTERT         ALGRR---KQAQHFTQRLKTLFSMLNYERTKHPHLMGSSVLGMNDIYRTWRAFVLRV-RA   689
TmtTERT       TLNRKSRDKKVHYFNTQLKNLFSVLNYERTENTSLLGSSVFGKDDIYKIWRKFVSEV-LE   918
PvTERT        TFSRKAGEKKIQYFNTQLKNLFSVLNYERIKNPSLLGSSVFGKDDIYAVWKKFVLKV-LK   895
xTERT         -QSKENQEKKIHHFSSQIRNLFSVLNYEWNRNCSLIGSSVFGMDDIYKKWKKFVLDF-EK   756
ZfTERT        --------DTLQYFQSCVRVLQNVLSVCVREAPGPMGSTVWGWQDIHRRLQDFSPQQ---   669
OlaTERT       --------SNTRLHHKRIRDLMSMLQARVRSAPALLGSTVWGMTDIHKVLRSLAPAQ---   653
TtTERT        N--------IKLNLNQILMDSQLVFRNLKDMLGQKIGYSVFDNKQISEKFAQFIEKW---   604
AtTERT        ----------------SLRDTHAVLKDIQLKEPDVLGSSVFDHDDFYRNLCPYLIHL-RS   677
Sc_Est2p      --EEFTIYKENHKNAIQPTQKI--LEYLRNKRPTSFTK-IYSPTQIADRIKEFKQRLLKK   516
```

```
AmexTERT      SNFKEKQYYFVKTDVTGAYDTIPHAKLIEVVSKILNPSD--NYCIRRYAEIWVDPAGHIR   973
hTERT         -QDPPPELYFVKVDVTGAYDTIPQDRLTEVIASIIKPQ--NTYCVRRYAVVQKAAHGHVR   756
mTERT         -LDQTPRMYFVKADVTGAYDAIPQGKLVEVVANMIRHSE-STYCIRQYAVVRRDSQGQVH   747
TmtTERT       SNDKIPRFYYVKADVTGAYDTIPHDKLVEVVSQILDPEKRTVYCIRRYAVIKITMSGQIR   978
PvTERT        LNAVLPRFYFVKADVIGAYDSIPHNKLVEVILQALSPDEKTTYSIRRYAVIIRTRNGLIR   955
xTERT         PQVEKLQFYFVKTDVKGAYDTIPHSKLDEVISKVINPNANEVYCIRRYATVSVDPTGRII   816
ZfTERT        -KSSPRPLYFVKVDVSGAYDSLPHLKLVEVLKEVLGPFAEQSFFLRQYSSVWSDPTRGLR   728
OlaTERT       -KDKPQPLYFVKVDVSGAYDSLPHDKLKEVITEALSPVQEEVFTVRHYAKIWADSHEGLK   712
TtTERT        KNKGRPQLYYVTLDIKKCYDSIDQMKLLNFFNQSDLI--QDTYFINKYLLFQRNKRPLLQ   662
AtTERT        QSGELPPLYFVVADVFKAFDSVDQGKLLHVIQSFLKDE----YILNRCRLVCCGK----R   729
Sc_Est2p      FNNALPELYFMKFDVKSCYDSIPRMECMRILKDALKNEN--GFFVRSQY-FFNTNTGVL-   572

AmexTERT      KSYKRHVATM----DDF---------LPNTKMFVS--------HLQQN------------   1000
hTERT         KAFKSHVSTL----TDL---------QPYMRQFVA--------HLQET------------   783
mTERT         KSFRRQVTTL----SDL---------QPYMGQFLK--------HLQDSDA----------   776
TmtTERT       KYYRRHVSTF----KDF---------MSHMRLFVS--------HLQES------------   1005
PvTERT        RYYRRHVSTY----KEF---------ISDMKEFIN--------HLQEV------------   982
xTERT         KSFKRHVSEL----ADV---------LPNMKQFVS--------NQQEK------------   843
ZfTERT        KRFCTKAEMS----E-----------PLNMKGFVV--------DEQVS------------   753
OlaTERT       KAFARQVDFS----DGSM-------GSTSMKGFVM--------SLQKS------------   741
TtTERT        IQQTNNLNSAMEIEEEKINKKPFKMDNINFPYYFNLKERQIAYSLYDDDDQILQKGFKEI   722
AtTERT        SNWVNKILVS----SDK---------NSNFSRFTS--------TVPYN------------   756
Sc_Est2p      KLFNVVN--A----SRV---------P---------------------------------   584

AmexTERT      -GSLKHAILVEQALHLNENSRNLLQFFQQMITNNILKVGDKCFVQCRGIPQGSILSTVLC   1059
hTERT         -SPLRDAVVIEQSSSLNEASSGLFDVFLRFMCHHAVRIRGKSYVQCQGIPQGSILSTLLC   842
mTERT         -SALRNSVVIEQSISMNESSSSLFDFFLHFLRHSVVKIGDRCYTQCQGIPQGSSLSTLLC   835
TmtTERT       -TSLQNAIVVEQSLSLNETSSSLFAFFLQMIHNNILEIEGRYYLQCCGIPQGSILSTLLC   1064
PvTERT        -TSLRNAVVVEQSVSLKESNTSLAEFFLKFIQNSILKIKDRYYVQRCGIPQGSILSTLLC   1041
xTERT         -NLLRNTILVEQNLLLNESSVKLLAVFQQIIRSHILRIKDRYYMQCCGIPQGSMLSTILC   902
ZfTERT        -GRLHDAILVER-HSSEVRGGDVFQFFQKMLCSYVIHYDQQMFRQVCGIPQGSSVSSLLC   811
OlaTERT       -SKVHHAVLVEQAFGSNLRGKDALQFFTQMLTGSVVQHGKKTYRQCRGIPQGSVVSSLLC   800
TtTERT        QSDDRPFIVINQDKPRCITKDIIHNHLKHISQYNVISFNKVKFRQKRGIPQGLNISGVLC   782
AtTERT        ---ALQSIVVDKGENHRVRKKDLMVWIGNMLKNNMLQLDKSFYVQIAGIPQGHRLSSLLC   813
Sc_Est2p      ---KPYELYIDNVRTVHLSNQDVINVVEMEIFKTALWVEDKCYIREDGLFQGSSLSAPIV   641

AmexTERT      SLCYGDMENNFFSGIQ---------------------QDGVFMRLIDDFLLVTPYLDKAE   1098
hTERT         SLCYGDMENKLFAGIR---------------------RDGLLLRLVDDFLLVTPHLTHAK   881
mTERT         SLCFGDMENKLFAEVQ---------------------RDGLLLRFVDDFLLVTPHLDQAK   874
TmtTERT       SLCYGDMENKLFCGIQ---------------------RDGVLIRLIDDFLLLTPHLTLAK   1103
PvTERT        NLCYGDMENKLLQGVQ---------------------KDGVLMRLTDDFLLVTPHLTQAK   1080
xTERT         SLCYGDMENAMLGGIQ---------------------KNGVLMRLIDDFLLVTPHLDQAK   941
ZfTERT        NLCYGHMEKALLKDIA---------------------KGGCLMRLIDDFLLITPHLSKAT   850
OlaTERT       CLCYGHMENVLFRDIK---------------------NKGCLMRLVDDFLLITPDRNQAQ   839
TtTERT        SFYFGKLEEEYTQFLKN--------------AEQVNGSINLLMRLTDDYLFISDSQQNAL   828
AtTERT        CFYYGHLERTLIYPFLEEASKDVSSKECSREEELIIPTSYKLLRFIDDYLFVSTSRDQAS   873
Sc_Est2p      DLVYDDLLEF-YSEFKA-----------------SPSQDTLILKLADDFLIISTDRQQVI   683

AmexTERT      TFLRTLAEGIPQYGCSISLSKTMVNFPLT-DLPGCSQAEQL---PARSVFPWCGVLFDTQ   1154
hTERT         TFLRTLVRGVPEYGCVVNLRKTVVNFPVEDEALGGTAFVQM---PAHGLFPWCGLLLDTR   938
mTERT         TFLSTLVHGVPEYGCMINLQKTVVNFPVEPGTLGGAAPYQL---PAHCLFPWCGLLLDTQ   931
TmtTERT       TFLRTLATGIPEYGFLINPKKTMMNFHVDEDIPGCSKFKQL---PYCGVIPWCGLLLDIQ   1160
PvTERT        MFLRTLAMGVPEYGFVINPAKTVVNFPIDEDIPGCSEFKQL---PAHGVIPWCGLLIDTQ   1137
xTERT         TFLRTLAEGIPQYGCSISPQKTVVNFPVD-DIPECSEVEQL---PSHCLFRWCGLLLDTQ   997
ZfTERT        EFLTTLLSGVPDYGCQINPQKVAVNFPVCVS-WVNSGVSVL---PSSCLFPWCGLMIHTH   906
OlaTERT       SFLSILLAGVPQYGVVANPQKVVVNFQGSEGGGAFPDIRVL---PPHCLFPWCGLLLDTR   896
TtTERT        NLIVQLQNCANNNGFMFNDQKITTNFQFPQEDYNLEHFKIS----VQNECQWIGKSIDMN   884
AtTERT        SFYHRLKHGFKDYNCFMNETKFCINFEDKEEH-RCSSNRMFVGDNGVPFVRWTGLLINSR   932
Sc_Est2p      NIKKLAMGGFQKYNAKANRDKILAVSSQS---------------DDDTVIQFCAMHIFVK   728

AmexTERT      TLEVYCDYSRYACTSIRSSLTISHKAETG-TSMRHKLIRVLKLKCKSIFLDLMVNSLRTV   1213
hTERT         TLEVQSDYSSYARTSIRASLTFNRGFKAG-RNMRRKLFGVLRLKCHSLFLDLQVNSLQTV   997
mTERT         TLEVFCDYSGYAQTSIKTSLTFQSVFKAG-KTMRNKLLSVLRLKCHGLFLDLQVNSLQTV   990
TmtTERT       TLEVYCDYSSYACTSIRSSLAFNSSNTAG-KNMKYKLVTVLKLKCHSLFLNLQVNSLRTV   1219
PvTERT        TLEVYCDYSSYACTSIRSSLSFNSSTKAG-VRLRNKLLAVLQLKCHSLFVDLQINSLRTV   1196
xTERT         TLDVYYDYSSYACTSIRSSMTFCHSSAAG-KYMKQKLIRVLRLKCHSLFLDLKVNSLRTV   1056
ZfTERT        TLDVYKDYSRYDGLSLRYSLTLGSAHSPS-TVM-KKLLSVLSIKSTDIFLDLRLNSVEAV   964
OlaTERT       SLDVCKDYSSYAGLSLRYSLTLGSAHSAG-QQMRRKLMSILRIKCHPLFLDLKTNSLESA   955
TtTERT        TLEIKSIQKQ-TQQEINQTINVAISIKNLKSQLKNKLRS-LFLNQLIDYFNPNINSFEGL   942
AtTERT        TFEVQVDYTRYLSGHISSTFSVAWQNKPV-RNLRQKLCYFLVPKCHPILFDSNINSGEIV   991
Sc_Est2p      ELEVWKHSSTMNNFHIRSKSS---------KGIFRSLIALFNTRISYKTIDTNLNSTNTV   779
```

```
AmexTERT      FINIYKIFLLQAYRFHACVIQL---PFN--------QGVRK-NPSFFLSVISDMAPCCYS   1261
hTERT         CTNIYKILLLQAYRFHACVLQL---PFH--------QQVWK-NPTFFLRVISDTASLCYS   1045
mTERT         CINIYKIFLLQAYRFHACVIQL---PFD--------QRVRK-NLTFFLGIISSQASCCYA   1038
TmtTERT       FINIYKIFLLQAYRFHACVLQL---PFN--------QQVRK-NPCFFLRIISDTASCCYS   1267
PvTERT        CINIYKILLLQAYRFHACVLQL---PFN--------QKIKS-NPRFFLKIISDTASCCFS   1244
xTERT         CINTYKIFLLQAYRFHACVVQL---PFG--------QRVMN-NPPFFLTVISDMAPCFYT   1104
ZfTERT        YRSLYKLILLQALRFHACVRSL---PLG--------QSVNR-NPSFFLKMIWRMTRVTNK   1012
OlaTERT       YKNIHKLVLLQACRFHVCVQSL---PFA--------QTVAK-NPTYFQQMIWDMAHYANA   1003
TtTERT        CRQLYHHSKATVMKFYPFMTKLFQIDLKKSKQYSVQYGKENTNENFLKDILYYTVEDVCK   1002
AtTERT        RLNIYQIFLLAAMKFHCYVYEV---SRF--------WKL---HPQTLFKFITISVRYMFR   1037
Sc_Est2p      LMQIDHVVKNISECYKSAFKDL---SIN--------VTQNMQFHSFLQRIIEMTVSGCP-   827

AmexTERT      ILKSKNIGIALGTKD-ACNTFPFEAAQWLSYHAFIVKLTCHKA---VYKCLLKPLQNCKV   1317
hTERT         ILKAKNAGMSLGAKG-AAGPLPSEAVQWLCHQAFLLKLTRHRV---TYVPLLGSLRTAQT   1101
mTERT         ILKVKNPGMTLKAS----GSFPPEAAHWLCYQAFLLKLAAHSV---IYKCLLGPLRTAQK   1091
TmtTERT       ILKTKNTGIALGTKG-ASGLFPSEAAEWLCYHAFIIKLTNHKV---VYKCLLRFLKICEL   1323
PvTERT        VLKTKNKDVSFTSTG-TSVVLTYEVVQWLTYHAFVIKLTNHKV---IYKCLLAPLKHCKK   1300
xTERT         TFKAKNKDLTRGYKD-VSCQFNFEAVQWLSYQAFLTKLHNHKV---LYKCLIGPLQNCKM   1160
ZfTERT        LLTHINKGLPVCSVD-SGGVLQSEAVQLLFCLAFETLFRRFRS---VYHCLIPALHKRKR   1068
OlaTERT       LIRRSNTGLVLGDGA-QKGSVQYEAVELLFCLAFLRVLSKHRP---VYKDLLPRLHKWKR   1059
TtTERT        ILCYLQFEDEINSNI---KEIFKNLYSWIMWDIIVSYLKKKKQFKGYLNKLLQKIRKSRF   1059
AtTERT        LINRRVRRINTGSSFRPVLKLYKEEVIWLGLDAYIQVLKKKNS---RYRMLLIYLKSALS   1094
Sc_Est2p      ----------IT----KCDPLIEYEVRFTILNGFLESLSSNTS---KFKDNIILLRKEIQ   870

AmexTERT      Q--------LSKKIHK-------ATIKMLNDVIEPS-----LHKDFAAILN---------   1348
hTERT         Q--------LSRKLPG-------TTLTALEAAANPA-----LPSDFKTILD---------   1132
mTERT         L--------LCRKLPE-------ATMTILKAAADPA-----LSTDFQTILD---------   1122
TmtTERT       Q--------LFKKIPK-------ATMQLLKTVTEPA-----LCKDFKAIMD---------   1354
PvTERT        R--------LLRQIPE-------TTVQFLKEVTEPS-----LYEDFKAILD---------   1331
xTERT         Q--------LSRRLSQ-------DTIELLKSVTDSS-----LHKDFSCIMD---------   1191
ZfTERT        A--------LQRELCG-------ITLARVRQASSPR-----IPLDFSMRV----------   1098
OlaTERT       R--------LERLLGD-------LRLARVRQAANPR-----ALLDFLAMQM---------   1090
TtTERT        FYLKEGCKSLQLILSQQKYQLNKKELEAIEFIDLNN-----LIQDIKTLIPKISAKSNQQ   1114
AtTERT        K------HSLSQQLSS-----------ELRYATDRSNSSSLWKLNY--------------   1123
Sc_Est2p      H--------LQAYIYI--------------YI----------------------------   880

AmexTERT      ---         1348
hTERT         ---         1132
mTERT         ---         1122
TmtTERT       ---         1354
PvTERT        ---         1331
xTERT         ---         1191
ZfTERT        ---         1098
OlaTERT       ---         1090
TtTERT        NTN         1117
AtTERT        ---         1123
Sc_Est2p      ---         880
```

Figure S1. Multiple Sequence Alignment (MSA) of TERT proteins of representative species: AmexTERT, *Ambystoma mexicanum*; hTERT, *Homo sapiens*; mTERT, *Mus musculus;* TmtTERT, *Terrapene mexicana triunguis*; PvTERT, *Pogona vitticeps;* xTERT, *Xenopus laevis*; ZfTERT, *Danio rerio;* OlaTERT, *Oryzias latipes;* Sc_Est2p, *Saccharomyces cerevisiae;* TtTERT, *Tetrahymena thermophila;* AtTERT, *Arabidopsis thaliana*; *HIV-1* RT.
