# Supplementary material for: An Identification and Characterization of the Axolotl (Ambystoma mexicanum, Amex) Telomerase Reverse Transcriptase (Amex TERT)
Source: Genes (Basel). 2022 Feb 18;13(2):373. doi: 10.3390/genes13020373 (PMC8924892; doi:10.3390/genes13020373)
Supplement: Supplementary file 1 [file genes-13-00373-s001.zip › genes-1577429 - supplementary/Figure S2.pdf]

Region v-I

```
                                                                          *            * * *
TERT con          M        VL  LRS Y          V  L  F              L            L Q G
AmexTERT      ----MQRGNDFAGVLRLLRSVYPQ--------VCTLIQFM---------PTFPV----VHGSATIQRSD   44
hTERT         ----MPRAPRCRAVRSLLRSHYRE--------VLPLATFV---------RRLG------PQGWRLVQRG   42
mTERT         ----MTRAPRCPAVRSLLRSRYRE--------VWPLATFV---------RRLG------PEGRRLVQPG   42
TmtTERT       ----MAGSGPFRAVLAMLEGCYAE--------VLGLEQFV---------QRLREAAACGPGEAELLQRG   48
PvTERT        ----MGRPRRWRAVQGLLRSCYAE--------VLPLEAFV---------ERLQGQAQAEGPLVPLVQEG   48
xTERT         ----MPLRTGGATLLSILQRLYGQ--------VLGIVEYT---------DTLQVP---GGIKVPVLLEG   45
ZfTERT        MSGQYSTDGGFRPVLEILRSLYPV--------VQTLEEFT---------DGLQFP---DGRKPVLLEET   49
OlaTERT       -----MTSGDLSSVLNILRSLYKR--------TRTLEEFA---------DGVVFR---EGRRAALLQPS   44
```

```
                                              **                           *   *
TERT con      D     R  V    VC        P    SF Q S   EVVARV QR CEK     NVLAFGY LLDE
AmexTERT      DGEHYRTFVSQLLVCIPGEAWPPPHSAKIQQLSSQREVVARVIQRICEK-QRSNVLAFGFALVDEN---   109
hTERT         DPAAFRALVAQCLVCVPWDARPPPAAPSFRQVSCLKELVARVLQRLCER-GAKNVLAFGFALLDGA---   107
mTERT         DPKIYRTLVAQCLVCMHWGSQPPPADLSFHQVSSLKELVARVVQRLCER-NERNVLAFGFELLNEA---   107
TmtTERT       DSELYQTFVSQCVVCVPRGARAIPQPLSFQQLSSESEVVIRVIQRLCEK-KKKNVLAFGYTLLDEN---   113
PvTERT        DPACYRHLVERCLVGRAPGCKALPSRVVFQQVSSLNDVVARVIQRICEK-KKKNLLASGYSLLNEN---   113
xTERT         DSEKFRSFVAELVVCIPRGTKPLPSPVSFLQLSTQREVVARVIQRICEK-KKKNVLAFGYGLVDEK---   110
ZfTERT        DGARFKKLLSGLIVCAYTPPQ-LR---VPAQLSTLPEVLAFTLNHIKRK-KLRNVLGFGYQCSDVT---   110
OlaTERT       DTHSFKKSFVRGVFVCSDEELQDVP---SCNQTCTFPELLAFILNSLKRK-RRRNVLAHGYNFLGVAQED   109
```

```
                           *                         *        *       *
TERT con           F       YLPN   T TI   S  LW   LL  R  GDDV   YLL    CA  F  LVPPSC  YQ  CG  P  Y
AmexTERT      SPM-PLTFAPNICNFLPNPTTATISTSVLWGTLLSRIGDDIMMYLLEHCSLFMLVPPTCCYQICGLPVY   177
hTERT         RGGPPEAFTTSVRSYLPNTVTDALRGSGAWGLLLRRVGDDVLVHLLARCALFVLVAPSCAYQVCGPPLY   176
mTERT         RGGPPMAFTSSVRSYLPNTVIETLRVSGAWMLLLSRVGDDLLVYLLAHCALYLLVPPSCAYQVCGSPLY   176
TmtTERT       SSYSQVMSTSNICSYLPNTATETLRMSVLWETLLSRIGDDVMMYLLEHCAIFMLVPPSCSYQICGQPVY   182
PvTERT        NYQ--LSCMPNIYTYLPNNTETIRQSILWEIILSRVGDDVMMYLLEHCALFMLVPPSCCYQMCGQPIY   180
xTERT         NSL-NIRLTPNICNYFPNPTTTTISTSILWETLLTRVGDDVMMYWLEQCSIFVFVPPRCCYQITGQPIY   178
ZfTERT        TSSDPFRFHGDV-----SQTAASISTSEVWKRINQRLGTEVTRYLLQDCAVFTTVPPSCVLQVCGEPVY   174
OlaTERT       RDADHFRFQGDL-----SQSAAYIHSSDLWKKVTARLGTDVTRYLLGSCSVFVLAPPSCIFQICGVPAY   173
```

Region v-II

```
                                   *
TERT con      LY        E    SFLLN L      G R      RLVE IFL
AmexTERT      LYS-NFSFREGFSVSFLLNTLKQCPFGGQ-----RLVENIFL  508
hTERT         LYS-SGDK-EQLRPSFLLSSLRPSLTGAR-----RLVETIFL  366
mTERT         LYS-RGDGQERLNPSFLLSNLQPNLTGAR-----RLVEIIFL  371
TmtTERT       LYS-SRSFRECFPKSFLLNRLKGCQAGGQ-----RLVETIFL  512
PvTERT        LYC-HRQLKERLPESFILNRLKSFSSGGR-----RLVEVIVL  501
xTERT         LYL-SISYKKGFSESFILNSLDSTPSGSQ-----KLVETIFL  414
ZfTERT        LYN-GRGM-----KNFLLNRKLKGVGGARRMQGEDLVRMIFL  382
OlaTERT       LYG-GRGM-----HGFCLNRKRRTAAGPRRLQGQDLVRLVFF  355
```

Region v-III

```
                    *     *
TERT con      LP RYW  M  P  FQ  LL  NH  KCPY    L    CPV
AmexTERT      LPKRYWPMKCVFQELVDNHNKCPYKVLLRNNCPVR   565
hTERT         LPQRYWQMRPLFLELLGNHAQCPYGVLLKTHCPLR   416
mTERT         LSRRYWQMRPLFQQLLVNHAECQYVRLLRSHCRFR   421
TmtTERT       LPRRYWQMRDVFQKLLKNHAKCPYLVLLKKNCPVW   569
PvTERT        LPKRYREMRDLFQELLENHSKCPYLKVLRKNCPVW   558
xTERT         LPKRYWKMKPHFQELIQNHKKFPYLVYLNKHCPVR   470
ZfTERT        LPKRFFAMVPLFSRLLRQHRKCPYRLFLQRKCAGN   428
OlaTERT       LPLRYFNMVPVFGRLLQRHRKCRYSSVLHRMCPVV   405
```

Region v-IV

```
                   *      *    *
TERT con           L   LL QHSS  WQVY  FVRECL   VVP   LWGS  HN   RFF  NVK  FIS  LGKF
AmexTERT      FRDSSFMKLLRQYSSLWQVYAFARECLKTVVPKELWGSNHNKYRFFKNVKTFIS-LGKFG   717
hTERT         TDPRRLVQLLRQHSSPWQVYGFVRACLRRLVPPGLWGSRHNERRFLRNTKKFIS-LGKHA   501
mTERT         TSPPHLMDLLRLHSSPWQVYGFLRACLCKVVSASLWGTRHNERRFFKNLKKFIS-LGKYG   491
TmtTERT       FCDPGLKELLRQHSSHWQVYVFVRECLDLVVPAELWGSSHNKCRFFKNVKTFIS-LGKFA   717
PvTERT        SSSSGVMTFLKQHSTHWQVYMFVRECIERVVPAALWGSSHNKCRFYKNVKKFIS-LGKFN   693
xTERT         TTKANLLSLLKQHSSIWQVYMFVRECLNNVVPDIMWGSSHNKCRFFRNVKSFLFFSGKFG   556
ZfTERT        -DVKDMESLLKSHSSPYRVYLFVRECLRHIIPHELWGCQENQLHFLSNVKNFLL-LGKFE   488
OlaTERT       -AQGELSSLIPQHCAPHRVYLFVRECLTAVVPEELWGSDHNRLQFFSRVRGFLK-SGKFE   469
```

```
                                                  *
TERT con        SL ELMWKM  V  DC  WLR   K      VPASEHR RE  ILAKF
AmexTERT      KFSLRELMWKMRVTDCAWLRLGK-DDHFVPASEHRFREVIFAKF   760
hTERT         KLSLQELTWKMSVRDCAWLRRSP-GVGCVPAAEHRLREEILAKF   512
mTERT         KLSLQELMWKMKVEDCHWLRSSP-GKDRVPAAEHRLRERILATF   502
TmtTERT       RFSLQELMWKMRVNDCTWLRVIK-GDHFVPALEHHYREEILAKF   728
PvTERT        VFSLRELMWKMRVKNCVWLRLNKEQGHFVPSSEHRFRQDLMAKF   705
xTERT         KISLSELMWSMRVEDCSWIRLQK-SDHFVPASEHLLREKILAKF   567
ZfTERT        RLTLVQLMWRMKVQACHWLGPKK-R---QCASEHRYREWMLGQC   495
OlaTERT       RISVAELMWKIKVMDCDWLKLRR-TAGRFPPSELAYRTRILSQF   479
```

Motif T

```
                                                          *
TERT con       WL    V  LLR FFY TE   QK  L FYRK  WSKL     R H
AmexTERT       WLMDTYVIQLLRSFFYITETMFQKNALFFYRKCIWSKLHKIGLGKHF----A  810
hTERT          WLMSVYVVELLRSFFYVTETTFQKNRLFFYRKSVWSKLQSIGIRQHL----K  594
mTERT          WLMDTYVVQLLRSFFYITESTFQKNRLFFYRKSVWSKLQSIGVRQHL----E  584
TmtTERT        WLMDTYIVQLLRSFFYITETMFQKNMLFFYRKLIWSKLQNIGMRNHF----A  810
PvTERT         WLMDSYVAELLRSFFYITETMFQKNLLFFFRKSIWSKLESIGIRNHL----A  787
xTERT          WLMDTYVIQLLKSFFYVTETMFQKHRLLFYRKSVWKKLQNIGLRKHL----E  649
ZfTERT         WMLSGFVVGLVRAQFYITESMGHKHTLRFYRGDVWSRLQDQAFRAHL----C  577
OlaTERT        WLLDGFVVGLVRACFYATESVGQKNAIRFYRQEVWSKLQDLAFRRHI----A  561
TtTERT         FIINKIVIPVLRYNFYITEKHKEGSQIFYYRKPIWKLVSKLTIVKLEEENLE  513
AtTERT         WLFLEIVKKLVHFNFYATESQGGRLNIYYYRKRSWERLISKEISKAL----D  592
Sc_Est2p       WLFRQLIPKIIQTFFYCTEISS-TVTIVYFRHDTWNKLITPFIVEYF----K  413
```

Motif 1

```
                                   +   °
TERT con                     S LRFIPK  G
AmexTERT       DVAALKQ---NPLISRLRFIPKPNG   842
hTERT          EVRQHREARPALLTSRLRFIPKPDG   629
mTERT          EVRHHQDTWLAMPICRLRFIPKPNG   619
TmtTERT        EIEKVQQKKCVPMASKLRFIPKPNG   845
PvTERT         EIKTLQQQKCVPLASKLRFIPKPNG   822
xTERT          ELENMQQWKNVPLVSRLRFIPKTNG   684
ZfTERT         QALKVPN---SAVTSRIRFIPKTSS   609
OlaTERT        QVASLPK---GTVISQLRFIPKTDG   593
TtTERT         -------SFQKYPQGKLRIIPKKGS   541
AtTERT         E-------SSRKKLSKFRFLPKANG   620
Sc_Est2p       NHNS--YTLSNFNHSKMRIIPKKSN   446
HIV-1          EGKISKIGPENPYNTPVFAIKKKDS   223
RT con                       p h hh K
```

Motif 2

```
                ° 	         +
TERT con        RPI
AmexTERT       LRPIVKVQNVLDVQLHSKEKRDRKMQYFN    871
hTERT          LRPIVNMDYVVGARTFRRE---KRAERLT    655
mTERT          LRPIVNMSYSMGTRALGRR---KQAQHFT    645
TmtTERT        LRPIVKVNSVSGAKTLNRKSRDKKVHYFN    874
PvTERT         LRPVVKQNGVVGAETFSRKAGEKKIQYFN    851
xTERT          LRPISKISSTLSSQ-QSKENQEKKIHHFS    712
ZfTERT         MRPITRLSGSR-----------DTLQYFQ    627
OlaTERT        MRPITRVIGAD-----------SNTRLHH    611
TtTERT         FRPIMTFLRKDKQKN--------IKLNLN    562
AtTERT         VRMVLDFSSSSRSQ---------------    634
Sc_Est2p       FRIIAIPCRGADE---EEFTIYKENHKNA    472
HIV-1 RT       WRKLVDFRELNKRTQDFWEVQLGIPHPAG    254
RT con         hR h       K
```

Motif A

```
                         °     °
TERT con            YFVK DV GAYD IP  KL EV      P
AmexTERT       KEKQYYFVKTDVTGAYDTIPHAKLIEVVSKILNP   952
hTERT          PPPELYFVKVDVTGAYDTIPQDRLTEVIASIIKP   735
mTERT          QTPRMYFVKADVTGAYDAIPQGKLVEVVANMIRH   725
TmtTERT        KIPRFYYVKADVTGAYDTIPHDKLVEVVSQILDP   955
PvTERT         VLPRFYFVKADVIGAYDSIPHNKLVEVILQALSP   932
xTERT          EKLQFYFVKTDVKGAYDTIPHSKLDEVISKVINP   793
ZfTERT         SPRPLYFVKVDVSGAYDSLPHLKLVEVLKEVLGP   705
OlaTERT        KPQPLYFVKVDVSGAYDSLPHDKLKEVITEALSP   689
TtTERT         GRPQLYYVTLDIKKCYDSIDQMKLLNFFNQSDLI   641
AtTERT         ELPPLYFVVADVFKAFDSVDQGKLLHVIQSFLKD   714
Sc_Est2p       ALPELYFMKFDVKSCYDSIPRMECMRILKDALKN   553
HIV-1 RT       LKKKKSVTVLDVGDAYFSVPLDEDFRKYTAFTIP   288
RT con               h  hDh  GY  h
                             AF
```

IFD

```
                                              *
TERT con         K     V        D             M  F           LQ
AmexTERT       IRKSYKRHVATM----DDF---------LPNTKMFVS--------HLQQN------------  1000
hTERT          VRKAFKSHVSTL----TDL---------QPYMRQFVA--------HLQET------------  783
mTERT          VHKSFRRQVTTL----SDL---------QPYMGQFLK--------HLQDSDA----------  776
TmtTERT        IRKYYRRHVSTF----KDF---------MSHMRLFVS--------HLQES------------  1005
PvTERT         IRRYYRRHVSTY----KEF---------ISDMKEFIN--------HLQEV------------  982
xTERT          IIKSFKRHVSEL----ADV---------LPNMKQFVS--------NQQEK------------  843
ZfTERT         LRKRFCTKAEMS----E-----------PLNMKGFVV--------DEQVS------------  753
OlaTERT        LKKAFARQVDFS----DGSM-------GSTSMKGFVM--------SLQKS------------  741
TtTERT         LQIQQTNNLNSAMEIEEEKINKKPFKMDNINFPYYFNLKERQIAYSLYDDDDQILQKGFKEI  722
AtTERT         -RSNWVNKILVS----SDK---------NSNFSRFTS--------TVPYN------------  756
Sc_Est2p       L-KLFNVVN--A----SRV---------P---------------------------------  584

TERT con          L     VEQ          L   F
AmexTERT       -GSLKHAILVEQALHLNENSRNLLQFFQQMITNNILKVGD  1039
hTERT          -SPLRDAVVIEQSSSLNEASSGLFDVFLRFMCHHAVRIRG  822
mTERT          -SALRNSVVIEQSISMNESSSSLFDFFLHFLRHSVVKIGD  815
TmtTERT        -TSLQNAIVVEQSLSLNETSSSLFAFFLQMIHNNILEIEG  1044
PvTERT         -TSLRNAVVVEQSVSLKESNTSLAEFFLKFIQNSILKIKD  1021
xTERT          -NLLRNTILVEQNLLLNESSVKLLAVFQQIIRSHILRIKD  882
ZfTERT         -GRLHDAILVER-HSSEVRGGDVFQFFQKMLCSYVIHYDQ  791
OlaTERT        -SKVHHAVLVEQAFGSNLRGKDALQFFTQMLTGSVVQHGK  780
TtTERT         QSDDRPFIVINQDKPRCITKDIIHNHLKHISQYNVISFNK  762
AtTERT         ---ALQSIVVDKGENHRVRKKDLMVWIGNMLKNNMLQLDK  793
Sc_Est2p       ---KPYELYIDNVRTVHLSNQDVINVVEMEIFKTALWVED  621
```

Motif B'

```
                   *
                   +       °     +   +  +
TERT con           Y Q  GIPQGS LS  LC LCYG ME
AmexTERT         KCFVQCRGIPQGSILSTVLCSLCYGDMENNFFSGI  1074
hTERT            KSYVQCQGIPQGSILSTLLCSLCYGDMENKLFAGI  857
mTERT            RCYTQCQGIPQGSSLSTLLCSLCFGDMENKLFAEV  850
TmtTERT          RYYLQCCGIPQGSILSTLLCSLCYGDMENKLFCGI  1079
PvTERT           RYYVQRCGIPQGSILSTLLCNLCYGDMENKLLQGV  1056
xTERT            RYYMQCCGIPQGSMLSTILCSLCYGDMENAMLGGI  917
ZfTERT           QMFRQVCGIPQGSSVSSLLCNLCYGHMEKALLKDI  826
OlaTERT          KTYRQCRGIPQGSVVSSLLCCLCYGHMENVLFRDI  815
TtTERT           VKFRQKRGIPQGLNISGVLCSFYFGKLEEEYTQFL  797
AtTERT           SFYVQIAGIPQGHRLSSLLCCFYYGHLERTLIYPF  828
Sc_Est2p         KCYIREDGLFQGSSLSAPIVDLVYDDLLEF-YSEF  655
HIV-1 RT         GIRYQYNVLPQGWKGSPAIFQSSMTKILEPFRKQN  329
RT con                   hPQG   pP hh   h
```

Motif C

```
                    + °°+
TERT con         L RL DDFLL TP
AmexTERT         FMRLIDDFLLVTPYLDK  1096
hTERT            LLRLVDDFLLVTPHLTH  879
mTERT            LLRFVDDFLLVTPHLDQ  872
TmtTERT          LIRLIDDFLLLTPHLTL  1101
PvTERT           LMRLTDDFLLVTPHLTQ  1078
xTERT            LMRLIDDFLLVTPHLDQ  939
ZfTERT           LMRLIDDFLLITPHLSK  848
OlaTERT          LMRLVDDFLLITPDRNQ  837
TtTERT           LMRLTDDYLFISDSQQN  826
AtTERT           LLRFIDDYLFVSTSRDQ  871
Sc_Est2p         ILKLADDFLIISTDRQQ  681
HIV-1 RT         IYQYMDDLYVGSDLEIG  350
RT con           h  F DDhhh
                    Y
```

Motif D

```
TERT con         A  FL  L  G P YG   N  K
AmexTERT         AETFLRTLAEGIPQYGCSISLSKTMV  1122
hTERT            AKTFLRTLVRGVPEYGCVVNLRKTVV  905
mTERT            AKTFLSTLVHGVPEYGCMINLQKTVV  898
TmtTERT          AKTFLRTLATGIPEYGFLINPKKTMM  1127
PvTERT           AKMFLRTLAMGVPEYGFVINPAKTVV  1104
xTERT            AKTFLRTLAEGIPQYGCSISPQKTVV  965
ZfTERT           ATEFLTTLLSGVPDYGCQINPQKVAV  874
OlaTERT          AQSFLSILLAGVPQYGVVANPQKVVV  863
TtTERT           ALNLIVQLQNCANNNGFMFNDQKITT  852
AtTERT           ASSFYHRLKHGFKDYNCFMNETKFCI  897
Sc_Est2p         VINIKKLAMGGFQKYNAKANRDKILA  708
HIV-1 RT         HRTKIEELRQHLLRWGLTTPDKKHQK  377
RT con                          Gh h  cK  h
```

Motif E

```
                       +° +
TERT con             PWCGLL
AmexTERT         RSVFPWCGVLF  1151
hTERT            HGLFPWCGLLL  935
mTERT            HCLFPWCGLLL  928
TmtTERT          CGVIPWCGLLL  1157
PvTERT           HGVIPWCGLLI  1134
xTERT            HCLFRWCGLLL  994
ZfTERT           SCLFPWCGLMI  903
OlaTERT          HCLFPWCGLLL  893
TtTERT           QNECQWIGKSI  881
AtTERT           VPFVRWTGLLI  929
Sc_Est2p         DTVIQFCAMHI  725
HIV-1 RT         EPPFLWMGYEL  388
RT con                 LG  h
                       M
```

CTE

```
                                         *     *                *  *
TERT con         DTQTLEVY DYSSYA TSIR SLTF S   AG  MR KL  VL LKCHSLFLDL VNSL TV
AmexTERT         DTQTLEVYCDYSRYACTSIRSSLTISHKAETGTSMRHKLIRVLKLKCKSIFLDLMVNSLRTV  1213
hTERT            DTRTLEVQSDYSSYARTSIRASLTFNRGFKAGRNMRRKLFGVLRLKCHSLFLDLQVNSLQTV  997
mTERT            DTQTLEVFCDYSGYAQTSIKTSLTFQSVFKAGKTMRNKLLSVLRLKCHGLFLDLQVNSLQTV  990
TmtTERT          DIQTLEVYCDYSSYACTSIRSSLAFNSSNTAGKNMKYKLVTVLKLKCHSLFLNLQVNSLRTV  1219
PvTERT           DTQTLEVYCDYSSYACTSIRSSLSFNSSTKAGVRLRNKLLAVLQLKCHSLFVDLQINSLRTV  1196
xTERT            DTQTLDVYYDYSSYACTSIRSSMTFCHSSAAGKYMKQKLIRVLRLKCHSLFLDLKVNSLRTV  1056
ZfTERT           HFHTLDVYKDYSRYDGLSLRYSLTLGSAHSPSTVM-KKLLSVLSIKSTDIFLDLRLNSVEAV  964
OlaTERT          DTRSLDVCKDYSSYAGLSLRYSLTLGSAHSAGQQMRRKLMSILRIKCHPLFLDLKTNSLESA  955

TERT con          INIYKI LLQAYRFHACV QLPF Q V KNP FFL  ISD A CCY
AmexTERT         FINIYKIFLLQAYRFHACVIQLPFNQGVRKNPSFFLSVISDMAPCCYS  1261
hTERT            CTNIYKILLLQAYRFHACVLQLPFHQQVWKNPTFFLRVISDTASLCYS  1045
mTERT            CINIYKIFLLQAYRFHACVIQLPFDQRVRKNLTFFLGIISSQASCCYA  1038
TmtTERT          FINIYKIFLLQAYRFHACVLQLPFNQQVRKNPCFFLRIISDTASCCYS  1267
PvTERT           CINIYKILLLQAYRFHACVLQLPFNQKIKSNPRFFLKIISDTASCCFS  1244
xTERT            CINTYKIFLLQAYRFHACVVQLPFGQRVMNNPPFFLTVISDMAPCFYT  1104
ZfTERT           YRSLYKLILLQALRFHACVRSLPLGQSVNRNPSFFLKMIWRMTRVTNK  1012
OlaTERT          YKNIHKLVLLQACRFHVCVQSLPFAQTVAKNPTYFQQMIWDMAHYANA  1003

                                         *                *
TERT con          LK KN G  LG          EAVQWL Y AF  KL  H V YKCLL  L
AmexTERT         ILKSKNIGIALGTKDACNTFPFEAAQWLSYHAFIVKLTCHKAVYKCLLKPLQNCKV  1317
hTERT            ILKAKNAGMSLGAKGAAGPLPSEAVQWLCHQAFLLKLTRHRVTYVPLLGSLRTAQT  1101
mTERT            ILKVKNPGMTLKAS---GSFPPEAAHWLCYQAFLLKLAAHSVIYKCLLGPLRTAQK  1091
TmtTERT          ILKTKNTGIALGTKGASGLFPSEAAEWLCYHAFIIKLTNHKVVYKCLLRFLKICEL  1323
PvTERT           VLKTKNKDVSFTSTGTSVVLTYEVVQWLTYHAFVIKLTNHKVIYKCLLAPLKHCKK  1300
xTERT            TFKAKNKDLTRGYKDVSCQFNFEAVQWLSYQAFLTKLHNHKVLYKCLIGPLQNCKM  1160
ZfTERT           LLTHINKGLPVCSVDSGGVLQSEAVQLLFCLAFETLFRRFRSVYHCLIPALHKRKR  1068
OlaTERT          LIRRSNTGLVLGDGAQKGSVQYEAVELLFCLAFLRVLSKHRPVYKDLLPRLHKWKR  1059

                    *                          *
TERT con          L R L   T   L     P L  DF  I D
AmexTERT         QLSKKIHKATIKMLNDVIEPSLHKDFAAILN  1348
hTERT            QLSRKLPGTTLTALEAAANPALPSDFKTILD  1132
mTERT            LLCRKLPEATMTILKAAADPALSTDFQTILD  1122
TmtTERT          QLFKKIPKATMQLLKTVTEPALCKDFKAIMD  1354
PvTERT           RLLRQIPETTVQFLKEVTEPSLYEDFKAILD  1331
xTERT            QLSRRLSQDTIELLKSVTDSSLHKDFSCIMD  1191
ZfTERT           ALQRELCGITLARVRQASSPRIPLDFSMRV-  1098
OlaTERT          RLERLLGDLRLARVRQAANPRALLDFLAMQM  1090
```

Figure S2. Comparison of different TERT motifs of representative species using MSA: Conserved amino acid residues are colored, similar amino acid residues are highlighted in yellow. Consensus sequence is shown at the highest row, RT consensus [35] is shown at the lowest row. Residues that have been shown to be essential for enzyme acitivity [36] are indicated by open circles (o), telomerase-specific signatures [34] are indicated by plus (+). Amino acid residues deviate from TERT consensus are marked by asterisk (*).
MSA of conserved regions among vertebrate TERTs of the N-terminal half (v-I to v-IV) [40].
MSA of RT motifs, motif T and the IFD. Motif T and the IFD are TERT-specific so that the alignment was performed without HIV-1 RT. Hydrophobic conserved residues in the IFD are highlighted in blue and charged/hydrophilic positions are highlighted in pink [37].
MSA of CTE. CTE-like regions are found in RTs and in TERTs, but significant sequence-similarities are conserved within vertebrate TERTs only.
