# Supplementary material for: An Identification and Characterization of the Axolotl (Ambystoma mexicanum, Amex) Telomerase Reverse Transcriptase (Amex TERT)
Source: Genes (Basel). 2022 Feb 18;13(2):373. doi: 10.3390/genes13020373 (PMC8924892; doi:10.3390/genes13020373)
Supplement: Supplementary file 1 [file genes-13-00373-s001.zip › genes-1577429 - supplementary/Figure S3.pdf]

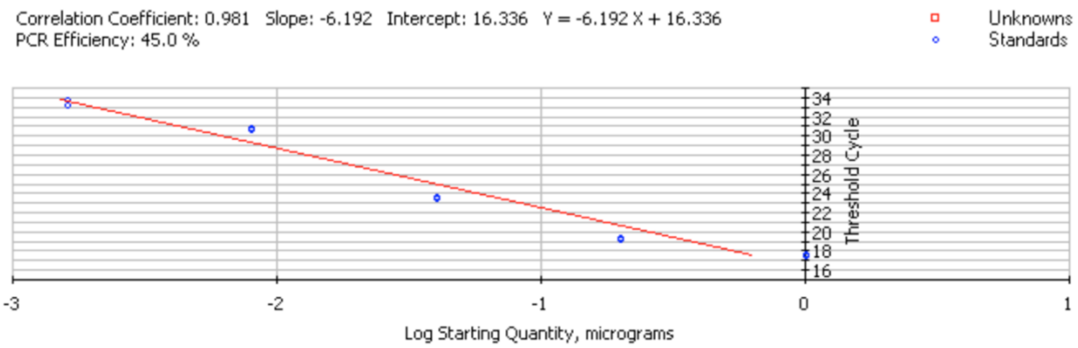

Figure S3. Determination of the standard curve and linear relationship for Q-TRAP. The Ct-values of the standard control were plotted against log[protein] to calculate the linear equation. They Y-intercept and the slope values from the equation are used to quantify the RTA of unknown samples ($10^{[(\text{Ct sample-Y-Int})/\text{slope}]}$).
