# Supplementary material for: An Identification and Characterization of the Axolotl (Ambystoma mexicanum, Amex) Telomerase Reverse Transcriptase (Amex TERT)
Source: Genes (Basel). 2022 Feb 18;13(2):373. doi: 10.3390/genes13020373 (PMC8924892; doi:10.3390/genes13020373)
Supplement: Supplementary file 1 [file genes-13-00373-s001.zip › genes-1577429 - supplementary/Table S1.pdf]

Table S1: RT-PCR raw data using primer TeloExp and adult limb mRNA respectively limb blastema mRNA. Every sample was applied three times and outliers were eliminated.

Well	Identifier	Ct	Mean Ct
A01	Blastema 1	30.8	30.7
A02	Blastema 1	30.6	
B01	Blastema 2	31.6	31.75
B02	Blastema 2	31.9	
C01	Blastema 3	29.6	29.6
C02	Blastema 3	29.6	
D01	Blastema 4	29.9	29.9
D02	Blastema 4	29.8	
D03	Blastema 4	30	
E01	Blastema 5	32.3	32.05
E03	Blastema 5	31.8	
F01	Blastema 6	28.4	28.57
F02	Blastema 6	28.6	
F03	Blastema 6	28.7	
G03	Limb 1	32.9	32.9
H03	Limb 2	33.4	33.4
A06	Limb 3	31.3	31.3
B06	Limb 4	32.7	32.7
C05	Limb 5	27.8	27.9
C06	Limb 5	28	
D05	Limb 6	30.3	30.4
D06	Limb 6	30.5	
E05	Limb 7	29.6	29.73
E06	Limb 7	30	
E07	Limb 7	29.6	
G05	Negative Control	N/A	
G06	Negative Control	N/A	
G07	Negative Control	N/A	
H07	Blank	N/A	
