# Supplementary material for: An Identification and Characterization of the Axolotl (Ambystoma mexicanum, Amex) Telomerase Reverse Transcriptase (Amex TERT)
Source: Genes (Basel). 2022 Feb 18;13(2):373. doi: 10.3390/genes13020373 (PMC8924892; doi:10.3390/genes13020373)
Supplement: Supplementary file 1 [file genes-13-00373-s001.zip › genes-1577429 - supplementary/Table S2.pdf]

Table S2: Primer sequences

Primer	Sequence (5´→ 3´)
Amex Tert	For: AAATGGTTTGCGCCCGATAGTC<br>Rev: ATAAAGGCATGGTAGCTAAGCCACTG
TeloExp	For: CCTCAAGCGTTTGTGTGTCA<br>Rev: TGGCTGCTCCATAAGCCTAA
Axolotl ornithin decarboxylase	For: CAGAATATTGGCGTGTCG<br>Rev: TTCCGTGCAACTTTCATC
Primer TS (Q-TRAP)	AATCCGTCGAGCAGAGTT
Primer ACX (Q-TRAP)	GCGCGGCTTACCCTTACCCTTACCCTAACC
M13	For: TGTAAAACGACGGCCAGT<br>Rev: CAGGAAACAGCTATGACC
